# Pulsed EPR Methods in the Angstrom to Nanometre Scale Shed Light on the Conformational Flexibility of a Fluoride Riboswitch

**DOI:** 10.1002/anie.202411241

**Published:** 2024-10-30

**Authors:** Laura Remmel, Andreas Meyer, Katrin Ackermann, Gregor Hagelueken, Marina Bennati, Bela E. Bode

**Affiliations:** ^1^ Research Group EPR Spectroscopy Max Planck Institute for Multidisciplinary Sciences Am Fassberg 11 37077 Göttingen Germany; ^2^ EaStCHEM School of Chemistry Biomedical Sciences Research Complex and Centre of Magnetic Resonance University of St Andrews North Haugh KY16 9ST St Andrews United Kingdom; ^3^ Institute of Physical Chemistry Georg-August University Tammannstraße 6 37077 Göttingen Germany; ^4^ Institute of Structural Biology University of Bonn Venusberg-Campus 1 53127 Bonn Germany

**Keywords:** ENDOR, PELDOR, DEER, fluorine, RNA

## Abstract

Riboswitches control gene regulation upon external stimuli such as environmental factors or ligand binding. The fluoride sensing riboswitch from *Thermotoga petrophila* is a complex regulatory RNA proposed to be involved in resistance to F^−^ cytotoxicity. The details of structure and dynamics underpinning the regulatory mechanism are currently debated. Here we demonstrate that a combination of pulsed electron paramagnetic resonance (ESR/EPR) spectroscopies, detecting distances in the angstrom to nanometre range, can probe distinct regions of conformational flexibility in this riboswitch. PELDOR (pulsed electron‐electron double resonance) revealed a similar preorganisation of the sensing domain in three forms, i.e. the free aptamer, the Mg^2+^‐bound *apo*, and the F^−^‐bound *holo* form. ^19^F ENDOR (electron‐nuclear double resonance) was used to investigate the active site structure of the F^−^‐bound *holo* form. Distance distributions without *a priori* structural information were compared with *in silico* modelling of spin label conformations based on the crystal structure. While PELDOR, probing the periphery of the RNA fold, revealed conformational flexibility of the RNA backbone, ENDOR indicated low structural heterogeneity at the ligand binding site. Overall, the combination of PELDOR and ENDOR with sub‐angstrom precision gave insight into structural organisation and flexibility of a riboswitch, not easily attainable by other biophysical techniques.

## Graphical abstract

A fluoride sensing riboswitch was investigated by EPR‐based distance measurements. Electron‐electron double resonance revealed preorganisation of the stem structures and conformational flexibility while electron‐nuclear double resonance demonstrates low structural heterogeneity around the fluoride site and distinct differences between the crystal and solution structures.

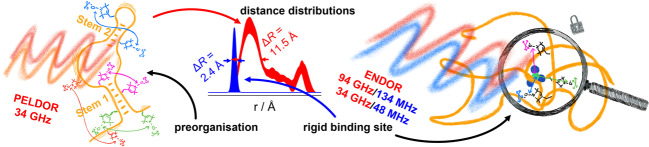

## Introduction

Protein structure and function underpins all aspects of life from the structural framework of cells, enzymatic activity, cellular transport, to immune function.^[1]^ The production of the individual proteins is controlled through the expression of the corresponding genes. Riboswitches are non‐coding RNAs mediating the regulation of genes[^2^, ^3^, ^4^, ^5^] by initiating or terminating transcription or translation. Here, gene expression is being triggered by environmental factors such as temperature or pH or through ligands like ions or small molecules.[^6^, ^7^, ^8^] Ion or metabolite sensing riboswitches form highly selective binding pockets for their specific target.^[9]^

Fluoride sensing riboswitches are present in both, bacteria and archaea.[^4^, ^10^, ^11^, ^12^] Increasing F^−^ concentrations can inhibit cell growth and become acutely toxic to cells.[^12^, ^13^, ^14^, ^15^, ^16^] This antimicrobial effect has been exploited to eliminate harmful microorganisms in fermentation processes.^[17]^ Fluoride riboswitches are involved in cellular defence mechanisms, such as initiating the production of F^−^ exporters.[^10^, ^12^, ^14^]

**Figure 1:**
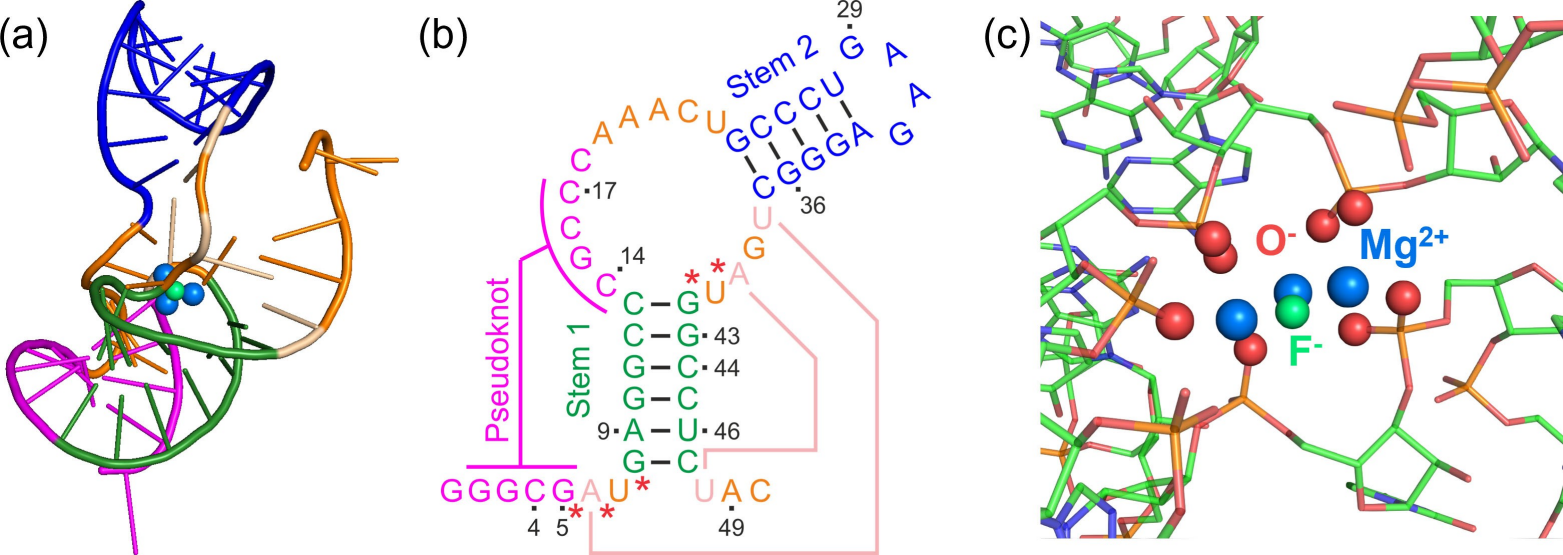
(a) Graphical representation of the crystal structure of the sensing domain of the fluoride binding riboswitch from *T. petrophila* in the *holo* form (PDB: 4ENC)^[4]^ with the F^−^ (green sphere) encapsulated by three Mg^2+^ (blue spheres); (b) schematic representation of the secondary structure of the 50 nucleotide construct of the fluoride binding riboswitch used here, with the two stem structures (green and blue), the pseudoknot structure (magenta), and the reversed Watson–Crick (A6⋅U38) and reversed Hoogsteen (A40⋅U48) base pairs (salmon). Residue numbers for spin‐labelling sites are indicated. The residues whose O^−^ are involved in the coordination of the Mg^2+^ are highlighted with red asterisks; (c) Graphical representation of the binding pocket of the fluoride binding riboswitch (PDB: 4ENC)^[4]^ with the F^−^ (green sphere) encapsulated by three Mg^2+^ (blue spheres). O^−^ involved in the coordination are represented as red spheres. Additional ions not involved in the fluoride encapsulation and water molecules have been omitted for clarity throughout.

The first fluoride sensing riboswitch was found in the *crcB* motif of *Pseudomonas syringae* in 2012.^[14]^ In the same year, the crystal structure of the sensing domain of the fluoride sensing riboswitch from the bacterium *Thermotoga petrophila* in its F^−^‐bound form was reported by Ren *et al*. (Figure 1a).^[4]^ The tertiary structure of the aptamer comprises two stems, a pseudoknot, and reversed Watson–Crick (A6⋅U38) and Hoogsteen (A40⋅U48) base pairs (Figure 1b). The riboswitch coordinates a cluster of three Mg^2+^ ions which in turn encapsulates the F^−^ ion (Figure 1c). In this way the polyanionic RNA avoids electrostatic repulsion with the bound F^−^. This motif composed of Mg^2+^, F^−^, phosphate, and water was computationally found to be stable largely based on the electrostatic interactions between F^−^ and Mg^2+^.^[18]^ A recent computational study proposes a stepwise assembly of the cluster with two Mg^2+^ bound in the *apo* form but the third Mg^2+^ only being incorporated together with F^−^.^[19]^ The riboswitch is strongly selective for F^−^ discriminating against other halides.^[4]^

Using solution nuclear magnetic resonance (NMR) spectroscopy, the fluoride binding riboswitch from *Bacillus cereus* was shown to adopt a highly similar fold.^[10]^ Here, stem 1 and stem 2 form already in absence of Mg^2+^ and F^−^,^[10]^ whereas the formation of the pseudoknot requires presence of Mg^2+^.[^20^, ^21^] Further addition of F^−^ does not result in a significant change of the tertiary structure but suppresses dynamics involving a lowly populated excited *apo* state responsible for transcription termination.[^10^, ^22^]

Pulsed electron‐electron double resonance (PELDOR,[^23^, ^24^] aka DEER for double electron‐electron resonance^[25]^) spectroscopy permits measuring distances ranging from 20–100 Å and beyond[^26^, ^27^, ^28^] between paramagnetic centres such as spin labels,[^26^, ^29^, ^30^, ^31^] paramagnetic metal ions,[^32^, ^33^, ^34^, ^35^, ^36^, ^37^] amino acid radicals and radical cofactors.[^38^, ^39^, ^40^] This provides a unique opportunity for investigating conformational ensembles through their spin‐spin distance distributions. Complementary to PELDOR, ^19^F electron‐nuclear double resonance (ENDOR) is an emerging technique to detect distances in a shorter range of about 5–20 Å between nitroxide spin labels and ^19^F nuclei.[^41^, ^42^, ^43^, ^44^] This has recently been expanded to distances between fluorine nuclei and other spin centres such as triarylmethyl (TAM) radicals,[^45^, ^46^] tyrosyl radicals,^[47]^ Cu^2+^,^[48]^ or Gd^3+^.[^49^, ^50^, ^51^]

In this work, we have investigated the *T. petrophila* riboswitch in solution by magnetic resonance methods. ^1^H NMR spectroscopy allowed monitoring the formation of base pairs through signals in the imino region of the free RNA, the Mg^2+^‐bound *apo* riboswitch, and the F^−^‐bound *holo* riboswitch. We have further studied the solution‐state preorganisation of the tertiary fold in the free and *apo* riboswitches by PELDOR and we have employed ^19^F ENDOR to investigate the structure of the binding pocket of the *holo* riboswitch.

## Results and Discussion

### Design of Spin Labelled RNAs

**Scheme 1:**
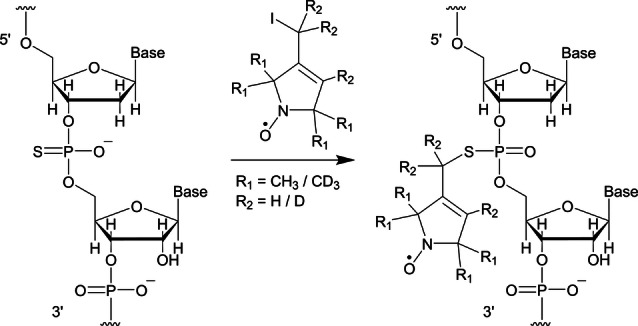
RNA labelling at the phosphorothioate modification of the backbone using either a protonated or a per‐deuterated spin label. The sugar 5’ to the labelling site is modified to a 2’‐deoxyribose to avoid strand scission.^[55]^

PELDOR and ^19^F ENDOR measurements require the presence of two or one paramagnetic spin labels, respectively. Several methods for site‐directed spin labelling of nucleic acids at the base, ribose, or phosphate exist.[^52^, ^53^] Custom synthesised RNA sequences with phosphorothioate modification are commercially available as is the spin label precursor for labelling at the phosphate backbone (Scheme 1) making this labelling approach readily accessible.[^54^, ^55^] This procedure leads to the introduction of a diastereomeric pair. The spin labelled RNA is therefore expected to display both diastereomers of the phosphothiotriester.

All constructs were designed based on the crystal structure of the *T. petrophila* riboswitch.^[4]^ The labelling sites were screened using *in silico* labelling with MtsslSuite^[56]^ and MMM^[57]^ to predict distances between pairs of spin labels or spin labels and F^−^. Furthermore, ensembles of spin label rotamers can also be extracted allowing analysis of orientation selection in the anisotropic EPR spectrum.^[25]^ In absence of an experimental high‐resolution structure a similar modelling can be applied using computational structure predictions that have become available also for nucleic acids with the introduction of AlphaFold3.^[58]^

To select the labelling sites, the following general criteria were applied: (i) nucleotides involved in the formation of the cluster, i.e. residues coordinating Mg^2+^ were excluded (red asterisks in Figure 1b); (ii) distances in the range between 18–45 Å were considered for PELDOR, and distances below 20 Å for ^19^F ENDOR.

For PELDOR measurements four different constructs with two spin labels each were prepared: G29/G36 within stem 2 and the corresponding hairpin (blue), C14/C44 located in the region between the pseudoknot and stem 1 (green), A9/A49 across stem 1 and the 3’ end of the RNA (magenta), and C4/C17 across the pseudoknot (red, Figure S5 and Figure S6).

For ^19^F ENDOR, three constructs with one spin label each were chosen to result in linearly independent distance vectors between the respective label and the F^−^, allowing the trilateration of the F^−^ position within the RNA structure (Figure S7). G5 and G43 gave respective calculated mean distances (*R*_model_) of 8.5 Å and 13.5 Å to the F^−^, well resolvable below the 20 Å threshold. For U46 however, *in silico* labelling displayed a bimodal distance distribution. The longer *R*_model,1_ was predicted to be at about 21.5 Å, likely too long to be resolved by ^19^F ENDOR. Nevertheless, the second, shorter *R*_model,2_ of 15.5 Å was within the resolvable distance limit for ^19^F ENDOR with nitroxide labels.^[41]^

To ensure reproducible folding of the riboswitch and infer any perturbation of folding by the spin label, we established a folding protocol (see Supporting Information section 1) using ^1^H NMR spectroscopy. This allowed us to identify the free aptamer in absence of Mg^2+^ and F^−^, the Mg^2+^‐bound *apo* riboswitch, and the F^−^‐bound *holo* riboswitch in analogy to results for the *B. cereus* fluoride riboswitch.^[10]^

Comparison of ^1^H NMR spectra in the imino regions of the free and *holo* aptamer for both, unlabelled and labelled RNA showed indistinguishable signals indicating appropriate folding after labelling (Figure S10–16).

Alternatively, isothermal titration calorimetry (ITC) could be used to investigate labelled and mutated riboswitch constructs for retained aptamer functionality.

### PELDOR Measurements

**Scheme 2:**
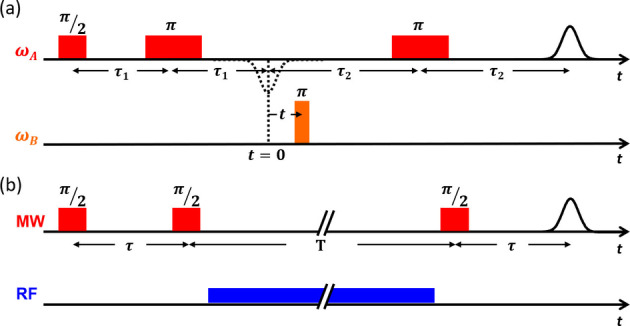
Pulse sequences for 4‐pulse DEER (a) and the Mims ENDOR (b) experiments with delays (t, τ), microwave (MW) pulses (π, π/2, red and orange), and a radiofrequency (RF) pulse (blue).

**Figure 2:**
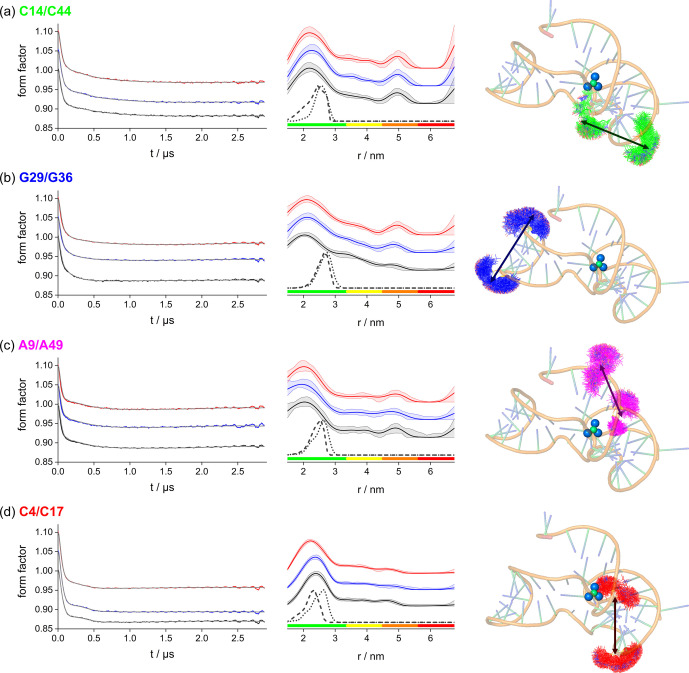
Background corrected PELDOR time traces (left) measured at Q‐band MW frequency and 50 K and analysed using Tikhonov regularisation for the free (black), *apo* (blue), and *holo* (red) aptamer labelled at positions C14/C44 (a), G29/G36 (b), A9/A49 (c), and C4/C17 (d). Sample concentrations were adjusted to 10 μM RNA (a‐c) and 20 μM RNA (d). The fit of the experimental time trace is given as grey line. Corresponding distance distributions (middle) with the respective 2σ confidence estimates. The colour bar is showing the reliability ranges (green: shape reliable, yellow: mean and width reliable, orange: mean reliable, red: no quantification possible) of the determined distributions. A vertical offset was introduced for both, the time traces and the distance distributions for clarity. The distance distributions obtained from *in silico* labelling with MtsslSuite (dashed line) and MMM (dotted line) are given for comparison. Graphical representation of RNA constructs (right, 4ENC^[4]^) spin labelled *in silico* with MtsslSuite and with arrows indicating the spin‐spin distance assed by PELDOR measurements.

PELDOR distance measurements using the 4‐pulse DEER sequence (Scheme 2 a) are particularly well suited to study structural or conformational changes.[^59^, ^60^, ^61^, ^62^] To map overall structure and preorganisation, PELDOR time traces were measured for the free, *apo* and *holo* aptamers for each of the four doubly spin labelled constructs. Sample preparation and experimental conditions for PELDOR are described in Supporting Information section 1. All spectra were recorded in frozen solution at 50 K. The time traces (Figure 2) displayed differently pronounced dipolar modulations, which indicated different widths of the respective distance distributions. The time traces were analysed using Tikhonov regularisation implemented in the software DeerAnalysis (Figure 2).^[63]^ All obtained distance distributions, despite being broad, showed a distinct most probable distance, but did not show significant differences between the free, *apo* and *holo* aptamers for the C14/C44, G29/G36, and A9/A49 constructs. The C4/C17 construct displayed the most pronounced dipolar modulation resulting in the narrowest distance distribution with the lowest uncertainty. Here, the free and *apo* forms provided no difference within uncertainty but the *holo* form yielded a slightly reduced most probable distance. These results could be confirmed by an alternative processing with a different implementation of Tikhonov regularisation in the software DeerLab^[64]^ and by neuronal network analysis with the software DeerNet^[65]^ (Figure S17). Interestingly, all distance distributions displayed significant distance probability up to 50 Å and beyond in all three processing approaches.

Comparing the experimental distance distributions with the modelling performed to select these constructs revealed substantially broader distributions in the former. Nevertheless, all modelled distance probability is fully covered by the experimental distributions. Particularly, the DeerLab analysis hinted at the presence of multiple distances corresponding to multiple conformations. This illustrated a conformational flexibility of the RNA backbone that was not reproduced by rotamer modelling on the single conformer in the crystal structure. Interestingly, for all cases but C4/C17 the most probable distances (i.e., maxima of the experimental distributions) were shorter than the maximum of the modelled distributions. This indicated the presence of more compact structural arrangements in solution than in the crystal structure. The similarity in the PELDOR data between the free, *apo*, and *holo* forms confirmed the preorganisation of stems 1 and 2 in the free RNA and their retention in the *apo* and *holo* aptamers. The shortening of the C4/C17 distance in the *holo* compared to the free and *apo* forms was consistent with an increased formation of the pseudoknot only upon addition of F^−^ in contrast to the findings for the *B. cereus* fluoride riboswitch.^[21]^ The long distances tailing up to 50 Å and beyond hinted to an equilibrium involving a disordered or unfolded form of the aptamer. This was consistent with earlier fluoride binding ITC data that had been fitted to 0.75 and 0.87 binding sites^[4]^ suggesting 13–25 % of aptamers not binding F^−^ and contributing very broad peaks to the distance distributions of all three forms.

### ^19^F ENDOR Spectroscopy

The fluoride binding riboswitch provides a unique opportunity to measure inter‐spin distances to the active site in the *holo* aptamer, containing an endogenous F^−^, by ^19^F ENDOR spectroscopy. For this, ^19^F Mims ENDOR (pulse sequence in Scheme 2b) was performed with three constructs containing a single nitroxide label at positions G5, G43, or U46 (Figure 1b). ^19^F ENDOR spectra of G5 and G43 were measured at W‐band (94 GHz) and four different excitation positions in the EPR spectrum (Figure S19, left). The orientationally selected ENDOR spectra were weighted by the spectral intensity at the excitation position, added, and analysed (see section 1 in Supporting Information).

For construct U46, spectra were measured at Q‐band (34 GHz), taking advantage of superior concentration sensitivity,^[46]^ as the expected distance was longer (Figure S20). At Q‐band, ^1^H ENDOR signals from the spin label methyl groups overlap with the ^19^F ENDOR signals (Figure S21). This was circumvented by introducing a deuterated nitroxide spin label (see section 1 in Supporting Information). Furthermore, this construct showed the slowest echo decay (i.e., slowest transverse dephasing, Figure S22) contributing to improved sensitivity. At 34 GHz ^19^F ENDOR spectra were measured only at two different spectral positions of the EPR line (Figure S19, right), and no significant effect of orientation selection could be observed within the achievable resolution and signal‐to‐noise ratio.

**Figure 3:**
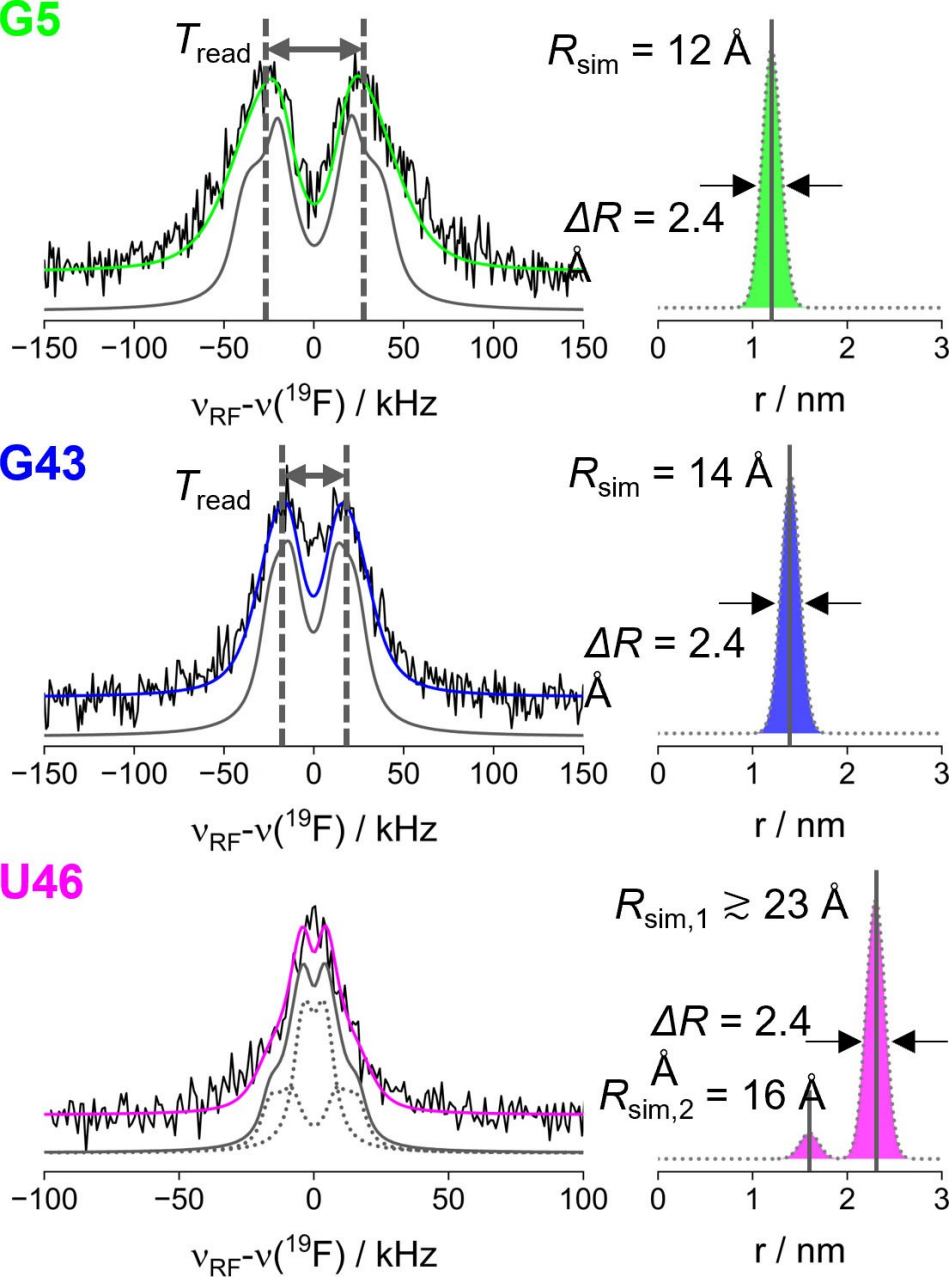
Mims ^19^F ENDOR sum spectra (left column) of the *holo* aptamer and simulated spectra (coloured lines) using Gaussian distributions (right column) or based on *T*_read_ (grey lines, left). In case of U46 the simulated spectrum contains contributions of two distance components (dotted grey). Mean distance (*R*_sim_) and FWH (Δ*R*) of the distance distributions used for simulations are given. Samples were composed of 100 μM, 150 μM, and 235 μM RNA for the RNA labelled in position G5, G43, and U46, respectively. Measurements were performed at W‐band (G5 and G43) and Q‐band (U46) and at 50 K. For W‐band, a protonated spin label and for Q‐band a deuterated one was used.

The three ^19^F ENDOR sum spectra of the *holo* riboswitch are displayed in Figure 3. For constructs G5 and G43, a well‐resolved hyperfine splitting arising from the maxima of a Pake pattern^[41]^ was observed. From this splitting, called *T*_read_, the perpendicular component *T*_⊥_ of the hyperfine tensor was estimated, giving first insight into the magnitude of the dipolar coupling and the corresponding inter‐spin distance. Using the point‐dipolar approximation the point‐dipole distance (*R*_read_) can be estimated as:^[41]^

(1) $$T_{read}\approx T_{⊥}=\frac{\mu_{0}}{4\pi h}\left(\frac{g_{iso}g_{n}\mu_{B}\mu_{N}}{R_{read}^{3}}\right)=\frac{C}{R^{3}}\;$$

with the vacuum permeability *μ*_0_, the Planck constant *h*, the *g*‐values of the nitroxide *g*_iso_=2.005 and the fluorine nucleus *g*_n_, the Bohr magneton *μ*_B_ and the nuclear magneton *μ*_N_. With these constants *C* amounts to *C*=74.52 MHz Å^3^.

For G5, a coupling constant *T*_read_=50±7 kHz was read off that corresponds (Eq. 1) to a point‐dipole distance of *R*_read_=11.5±0.5 Å. The error was estimated from the width of the maxima of the peaks. For G43, a smaller splitting from a coupling constant *T*_read_=30±4 kHz was observed, corresponding to a point‐dipole distance of 13.5±0.6 Å. For U46 no splitting could be observed, however a clear peak was still detected. To ensure that this signal of U46 did not arise from free F^−^ interacting with the nitroxide label, a control experiment was performed. ^19^F ENDOR spectra of the *free* aptamer labelled in position G43 and U46 were measured in the presence of F^−^ at W‐band and Q‐band, respectively. In absence of Mg^2+^ the F^−^ binding site cannot form and indeed no ENDOR effect has been observed (Figure S23). Thus, all ^19^F ENDOR signals observed in the *holo* form arose from bound F^−^.

To extract more information on conformational distributions from the ENDOR line shape we performed a spectral analysis in two steps. First, we simulated the spectra using the fast simulation routine SimSpec^[43]^ (see section 1 in Supporting Information) without any prior model, just assuming a Gaussian distribution of distances centred at *R*_read_. The centre and the width of the Gaussian distribution were manually adjusted to minimize the residual between experiment and simulated spectrum (Figure S25 and S26). The simulations are displayed in Figure 3 (left). The Gaussian distance distribution with a full width at half maximum (FWHM) of 2.4 Å reproduced the line shapes of G5 and G43 very well. The simulated *T*_sim_ component resulted in *R*_sim_ values as given in Figure 3 (right) and in Table 1. For U46 a series of simulations was performed for Gaussian distributions of constant width 2.4 Å but varying *R*_sim_ between 18 and 28 Å. Examination of the residuals (Figure S27 and Figure S28) suggested that *R*_sim_ ≳ 20 Å can generally be considered a lower boundary for the distance, when no splitting occurs. We note that this is the first detection of a ^19^F‐nitroxide distance ≳ 20 Å, demonstrating an extension of the accessible distance range. Indeed, the resolution of the splitting depended on the choice of the line width parameter. Our choice of a Lorentzian line of 7 kHz (slightly larger than the power broadening of the RF pulse) is based on an ongoing investigation of intrinsic ENDOR line widths. This resulted in *R*_sim_ ≳ 23 Å. Using a larger line width of 12 kHz, resulted in a shorter limit of *R*_sim_ ≳ 21 Å (Figure S28). Moreover, a single distance above 20 Å was not sufficient for reproducing the broad base of the ENDOR spectrum. This feature could be simulated by introducing an additional, shorter distance centred at about 16 Å and in a much smaller population (only 10 % of the overall distance distribution). We note that the Mims ENDOR experiment enhances the contribution of shorter distances, making such a small contribution discernible in the spectrum. In order to examine to what extent conformational distributions affect the spectra, we also performed simulations of rigid Pake patterns based on a single *R*_sim_ value convoluted with the estimated intrinsic ENDOR line width. Comparison of spectral simulations (Figure 3, Figure S29) showed indeed additional broadening caused by a distance distribution between F^−^ and spin label, which was most pronounced for the shortest distance in G5.

**Table 1:** Summary of the main contributions to the dipolar *T*, *R*, and Δ*R* parameters and uncertainties obtained for samples labelled at G5, G43 and U46 from the read‐off spectra, the simulation with Gaussian distributions of distances, and simulations with the rotamer model as well as the line width (LW) used for simulations. Errors of *T*_read_ and *R*_read_ were estimated from the width of the maxima of the peaks in the spectra. Errors of *T*_sim_ and *R*_sim_ were estimated based on simulations with different *T*_sim_/*R*_sim_.

Sample	*T*_read_/kHz	*T*_sim_/kHz	*R*_read_/Å	*R*_sim_/Å	*ΔR*_sim_/Å	*R*_model_/Å	*ΔR*_model_/Å	*LW/*kHz
**G5**	50±7	43∈[41, 53]	11.5∈[10.9, 12.0]	12∈[11.2, 12.2]	2.4	8.5	2.1	14
**G43**	30±4	27∈[25, 30]	13.5∈[13.0, 14.2]	14∈[13.6, 14.4]	2.4	13.5	2.2	14
**U46**	≲ 20	≲ 6	>15	≳ 23	2.4	21.5	2.4	7

**Figure 4:**
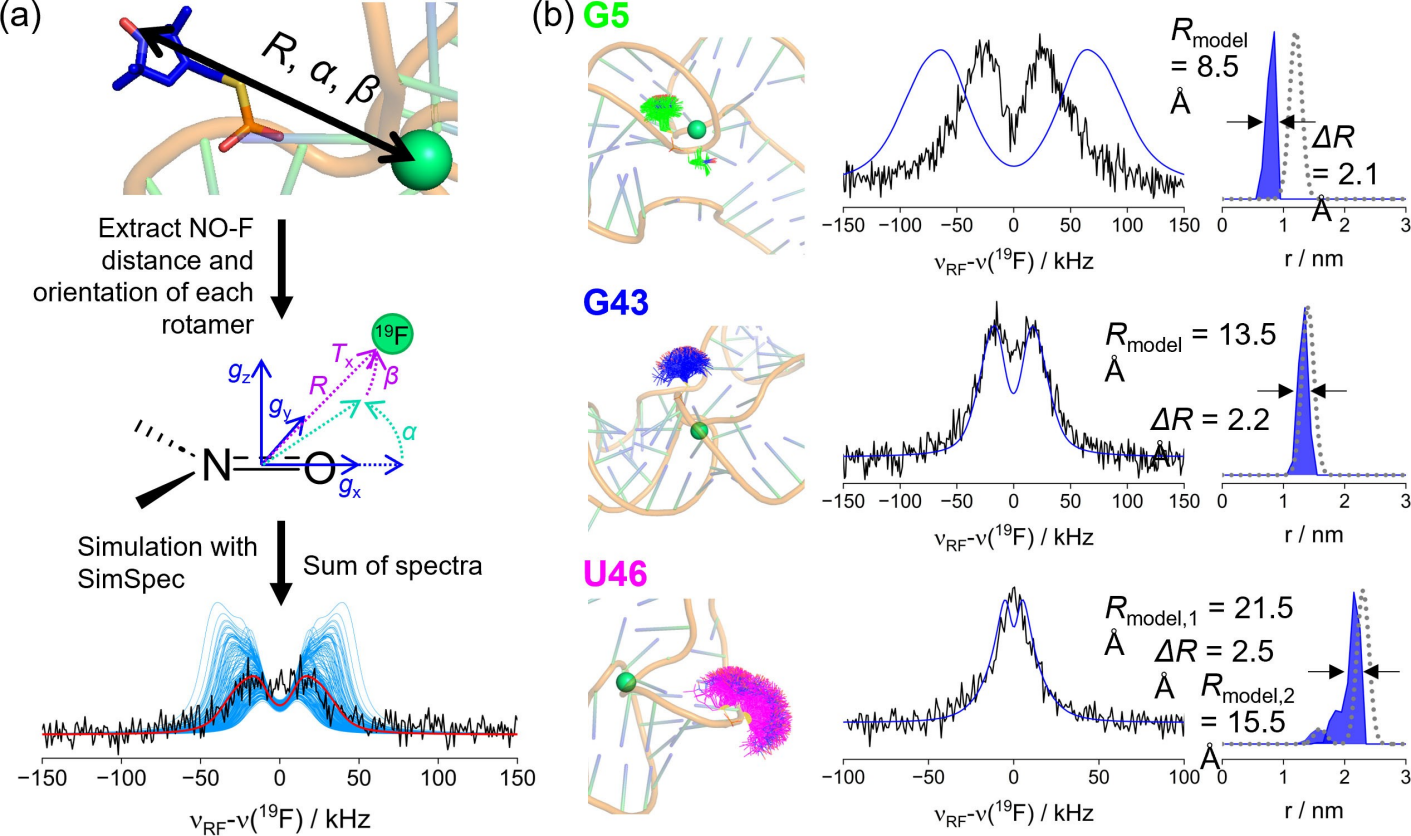
(a) Schematic representation of the workflow for the simulation of ^19^F ENDOR spectra based on the crystal structure spin labelled *in silico*. Distances (R) and orientations (α, β) of all rotamers were extracted, a ^19^F ENDOR spectrum for each rotamer was simulated using SimSpec^[43]^ and the sum of the simulated spectra was built; (b) Graphical representation of the fluoride riboswitch constructs labelled in position G5, G43, or U46 (left) with the respective Mims ^19^F ENDOR sum spectra (centre, black) and simulated spectra (centre, blue) using the distance distributions from *in silico* spin labelling (right). Mean distance (*R*_model_) and FWHM (Δ*R*) of the distance distributions from *in silico spin* labelling are given. The Gaussian distribution of distances is plotted as grey dotted line for comparison.

In the second step of analysis, we estimated the contribution of the spin label to the ENDOR line width by rotamer modelling of label orientations in MtsslSuite and MMM (Figure S8), based on the available crystal structure (PDB: 4ENC, see section 1 in Supporting Information). This modelling produces rotamer ensembles of spin label orientations as represented in Figure 4b. For each rotamer, distance and orientation were considered. The distance was taken from the midpoint of the NO‐bond to the F^−^ while the orientation was defined through the Euler angles α and β between the dipolar tensor and the spin label *g*‐tensor, as illustrated in Figure 4a. The Mims ENDOR spectrum for each of up to 400 rotamers was computed in SimSpec^[43]^ (see section 1 in Supporting Information) and then added to generate the sum ENDOR spectrum (Figure 4b). Both diastereomers were considered, however the modelling does not predict their relative weight. For G5, both diastereomers were considered in equal weight, while for G43 only one diastereomer was considered, as the second cannot be populated due to steric clashes. For U46, the weight of the diastereomer leading to the shorter distance was adjusted to reproduce the ENDOR spectrum.

Starting from the construct labelled in position G5 we obtained a substantial deviation of 3 Å between the predicted *R*_model_ and the observed *R*_sim_ distance. However, the distance distribution width with *ΔR=*2.1 Å was very close to the simulated value of 2.4 Å (Figure 4b). For construct G43 we found a good agreement between the Gaussian and the predicted distance distribution. For U46 a small deviation in the long distance (*R*_model,1_) could be observed while the width of this distribution contribution was in good agreement with the Gaussian.

For all three constructs, we found that the width of the distance distributions predicted by the rotamer modelling, when keeping a rigid body of the RNA, was consistent with the width extracted from the model‐free Gaussian distributions. This means that no substantial broadening arising from RNA backbone heterogeneity is observed in the ENDOR spectra. This is a significant result, which differed from the PELDOR findings, where the experimental distance distributions were considerably broader than the ones predicted from rotamer modelling. This is important, as it is known that rigid biomolecules (e.g., model protein GB1) allow reconciling PELDOR and RIDME data[^66^, ^67^] as well as ENDOR data^[50]^ all based on a single protein structure.

Furthermore, considering the mean distances, we observed good agreement only for G43 and U46. At G5, the significant deviation between the experimental and modelled distances can be rationalised by crystal contacts in the 5’ region (Figure S32) not being present in solution (see below). We conclude that the ENDOR data, although sensitive to small distance shifts of a few angstroms, do not report structural heterogeneity of the *holo* aptamer in the vicinity of the binding pocket. This indicates that the RNA structure around the active site is well‐defined in the *holo* form.

The observed deviations between the modelled and experimental ^19^F ENDOR distances around G5 prompted us to trilaterate the F^−^ position based on the experimental distances. This places the F^−^ in a position between the pseudoknot and the unpaired bases between the pseudoknot and stem 2 (Figure S31a). Here, the F^−^‐Mg^2+^ cluster could not be stabilised by coordination from the phosphate backbone (Figure S31b), strongly indicating a different structure or conformation around G5 well beyond the error of the ^19^F ENDOR measurement. The crystal structure shows significant intermolecular contacts (Figure S32) for the first two bases from the 5’ end, suggesting a structural variation in absence of these contacts in solution. The deviation in the distance between the nitroxide spin label and the F^−^ at G5 could be explained by a different orientation of the first five nucleotides at the 5’ end. The distance between the G5 phosphate group and the F^−^ is 6.3 Å in the crystal structure. The spin label rotamers have distances of 7–7.5 Å between phosphate and NO group adding to a maximum NO−F distance of about 14 Å. Thus, the 12 Å distance seen in the ^19^F ENDOR measurement (Figure 3) could arise from a differently oriented phosphate group. Nevertheless, a high similarity between five crystal structures of the riboswitch in presence of various ions^[4]^ as well as with the NMR derived structure of the *B. cereus* riboswitch^[10]^ has been reported (Figure S33).

Thus, the ENDOR and PELDOR results reveal different levels of structural heterogeneity—representing different functional dynamics—not directly obvious from crystal structures.

## Conclusion

In this work we have shown that the free, *apo* and *holo* forms of the sensing domain of the fluoride riboswitch from *T. petrophila* could be reproducibly formed in vitro. However, a significant population of unfolded or disordered aptamer was present in all PELDOR samples manifesting as distance distributions tailing up to 50 Å and beyond. Nevertheless, PELDOR has revealed a preorganisation similar to the *B. cereus* fluoride riboswitch with the exception that also F^−^ was required for pseudoknot formation.^[10]^ Modelling the widths of distance populations based on the single backbone conformation of the crystal structure matched the experimental distributions for ^19^F ENDOR but significantly underestimated the experimental PELDOR distance distributions. Thus, the combined PELDOR and ^19^F ENDOR results indicated a preorganised riboswitch structure with a more rigid F^−^‐binding site and more conformations sampled at the periphery.

For further understanding of conformational flexibility and presence of unfolded forms, investigation of the fluoride riboswitch structure in solution using, e.g., NMR or fluorescence methods is needed. Molecular dynamic simulations are complicated by the unusual cluster of Mg^2+^ ions, but might lead to further insight.[^18^, ^19^]

As a next step, extending the riboswitch from the sensing domain to the full‐length expression platform will potentially allow forming the transcription terminator state in the absence of F^−^ and observing the inherent conformational change by EPR methods, giving further insights into this intricate molecular mechanism of gene regulation.

Finally, we have shown that the use of a fluorine bearing ligand allows to selectively probe the ligand bound form by ^19^F ENDOR in a complex mixture of bound and unbound molecules. In a more general context, similar strategies could be employed to study ligand binding of fluorinated pharmaceuticals to biological targets.

^19^F ENDOR complements PELDOR with more precise distances on a shorter length scale. Here, we first observed a F^−^‐nitroxide distance in the range of 20 Å, which completely closes the gap in the distance range accessible from pulse dipolar spectroscopies. Therefore, the combination of the two EPR‐based methods, for distance measurements in frozen solution under similar sample conditions, provides valuable information on structure and conformational distributions, constituting a powerful tool for validation of structural models of complex biological systems.

## Supporting Information

The authors have cited additional references within the Supporting Information.[^68^, ^69^, ^70^, ^71^, ^72^, ^73^, ^74^, ^75^, ^76^, ^77^, ^78^]

Original spectroscopic data and codes are available in the Göttinger Research Online Data Base https://doi.org/10.25625/OPALQN.

Original spectroscopic data and codes are available in the Göttinger Research Online Data Base https://doi.org/10.25625/OPALQN.

## Conflict of Interests

The authors declare no conflict of interest.

## Supporting information

As a service to our authors and readers, this journal provides supporting information supplied by the authors. Such materials are peer reviewed and may be re‐organized for online delivery, but are not copy‐edited or typeset. Technical support issues arising from supporting information (other than missing files) should be addressed to the authors.

Supporting Information
