# Supplementary material for: Pulsed EPR Methods in the Angstrom to Nanometre Scale Shed Light on the Conformational Flexibility of a Fluoride Riboswitch
Source: Angew Chem Int Ed Engl. 2024 Oct 30;63(49):e202411241. doi: 10.1002/anie.202411241 (PMC11586693; doi:10.1002/anie.202411241)
Supplement: Supplementary file 1 — Supporting Information [file ANIE-63-e202411241-s001.pdf]

Angewandte Chemie
Eine Zeitschrift der Gesellschaft Deutscher Chemiker

Supporting Information

Pulsed EPR Methods in the Angstrom to Nanometre Scale Shed Light on the Conformational Flexibility of a Fluoride Riboswitch

L. Remmel, A. Meyer, K. Ackermann, G. Hagelueken, M. Bennati*, B. E. Bode**

Pulsed EPR Methods in the Angstrom to Nanometre Scale Shed Light on the Conformational Flexibility of a Fluoride Riboswitch – Supporting Information

Laura Remmel,[a,b] Andreas Meyer,[a,c] Katrin Ackermann,[b] Gregor Hagelueken,[d] Marina Bennati,[a,c] Bela E. Bode[b]

[a] Dr Laura Remmel, Dr Andreas Meyer, Prof. Dr Marina Bennati
Research Group EPR Spectroscopy
Max Planck Institute for Multidisciplinary Sciences
Am Fassberg 11, 37077 Göttingen, Germany
E-mail: andreas.meyer@mpinat.mpg.de

[b] Dr Laura Remmel, Dr Katrin Ackermann, Dr Bela E. Bode
EaStCHEM School of Chemistry, Biomedical Sciences Research Complex and Centre of Magnetic Resonance
University of St Andrews
North Haugh, St Andrews, KY16 9ST, United Kingdom
E-mail: beb2@st-andrews.ac.uk

[c] Dr Andreas Meyer, Prof. Dr Marina Bennati
Institute of Physical Chemistry
Georg-August University
Tammannstraße 6, 37077 Göttingen, Germany

[d] Dr Gregor Hagelueken
Institute of Structural Biology
University of Bonn
Venusberg-Campus 1, 53127 Bonn, Germany

Contents

1. Materials and Methods 1
1.1. Preparation of buffers 1
1.2. Folding protocol 1
1.3. *in silico* spin labelling 1
1.4. Trilateration of the fluoride position 2
1.5. Synthesis of the deuterated spin label 3
1.6. Labelling and purification of the fluoride riboswitch 6
1.7. Concentration determination 6
1.8. Sample preparation for spectroscopy 6
1.9. NMR measurements 7
1.10. EPR measurements and data analysis 8
1.10.1. PELDOR measurements 8
1.10.2. ^{19}F ENDOR measurements 9
2. NMR characterisation of unlabelled samples 11
2.1. ^{1}H NMR analysis at room temperature 11
2.2. ^{1}H NMR analysis at decreased and elevated temperatures 13
3. Selection of labelling sites 15
3.1. Selection of labelling sites for PELDOR measurements 15
3.2. Selection of labelling sites for ^{19}F ENDOR measurements 18
4. Analysis of the spin-labelled fluoride riboswitch constructs 22
5. Control Experiments and analysis for PELDOR 28
5.1. PELDOR analysis on doubly labelled constructs 28
5.2. PELDOR measurements using a constructs with one spin label 29
6. Control Experiments and analysis for ^{19}F ENDOR 30
6.1. Echo detected EPR measurements 30
6.2. ^{19}F ENDOR measurements of U46 at W-band 31
6.3. ^{19}F ENDOR measurements at Q-band with protonated spin label 31
6.4. T_M measurements 32
6.5. Background measurement 33
6.6. ^{19}F ENDOR measurements at different field positions 34
6.7. Estimation of coupling constant and width of Gaussian distribution of distances 34
6.8. Analysis with rotamers from MtsslSuite and MMM 38
7. Analysis of the riboswitch structure 39
References 41

1. Materials and Methods

1.1. Preparation of buffers

All buffers and solutions (1 M potassium acetate (KOAc) pH 6.1, 100 mM magnesium acetate ($Mg(OAc)_2$), 100 mM potassium fluoride (KF)) were prepared separately in diethyl pyrocarbonate (DEPC) treated and autoclaved MilliQ® water. In case of a pH adjustment this was done using potassium hydroxide and acetic acid solutions. All buffers were filtered through sterile 0.2 µm syringe filters. For deuteration the buffer solution was freeze-dried and re-dissolved in DEPC treated and autoclaved D_2O. Dilutions of the stock solutions were prepared using DEPC treated H_2O or D_2O as required.

1.2. Folding protocol

The folding of the fluoride riboswitch was performed according to the following protocol. Freeze dried RNA was dissolved in DEPC treated H_2O or D_2O. Previously dissolved frozen RNA was thawed on ice. 50 mM KOAc pH 6.1 was added, the RNA was heated to 95 °C for 10 min and annealed on ice for 10 min afterwards. The riboswitch was diluted with buffer and $Mg(OAc)_2$ and KF were added as required.

1.3. *in silico* spin labelling

The *in silico* spin labelling of the fluoride riboswitch was performed using the software applications MtsslSuite[1–3] and MMM.[4,5]

Since the introduction of a phosphorothioate modification to the RNA backbone leads to the presence of two diastereomers the possible rotamers were calculated for both diastereomers of the fluoride riboswitch. While this is already implemented in MMM the diastereomeric labels had to be selected separately in MtsslSuite. The label used for this is accessible as 'bebRNA1' and 'bebRNA1diast' in MtsslSuite and as R5-TP in MMM. In addition, the respective distance distribution was calculated, either between the spin labels of two labelling sites in case of pulsed electron-electron double resonance (PELDOR) measurements or between the spin label at the selected labelling site and the F^- for ^{19}F electron-nuclear double resonance (ENDOR) measurements. For PELDOR measurements two labelling sites were selected for each construct and for ^{19}F ENDOR measurements one labelling site was picked for each construct. Residues A6, U7, G8, U41 and G42 were excluded from the possible labelling sites as those residues are involved in the coordination of the F^-.

For the analysis of ^{19}F ENDOR data the separate rotamers were extracted from the output for both software applications and the orientation and distance towards the F^- was determined for each rotamer. In addition to the rotamers themselves MMM gives a population factor for each of the rotamers. Those population factors were extracted, correlated to the according rotamer.

1.4. Trilateration of the fluoride position

The trilateration of the F^- position was performed with MtsslTrilaterate within MtsslSuite.[1–3] The spin label conformers as anchor points were loaded from the MtsslSuite output. The mean distances determined from the ^{19}F ENDOR measurements and the full width at half maximum (FWHM) of the respective distribution used for the simulation of the ^{19}F ENDOR spectra were set as distance and width for the trilateration, respectively (Table S1). The trilateration was performed with a trial atom sphere size of 100 Å, 1,000,000 trials, and a cut-off value for the probability cloud (χ^2) of 0.5.

Table S1: Parameters used for the trilateration of the F^- with MtsslTrilaterate. Given are the labelling site, the diastereomer, and the mean distance and FWHM from the distance distribution used for simulation of the ^{19}F ENDOR spectra.

Labelling site	Spin label diastereomer	Mean distance/Å	FWHM/Å
G5	bebRNA	11.2	2.4
	bebRNAdiast	11.2	2.4
G43	bebRNA	13.2	2.0
U46	bebRNAdiast	21.0	2.1

1.5. Synthesis of the deuterated spin label

Synthesis of the spin label precursor was performed adapted from the literature.[6–10]

Synthesis of 4-deutero-3-(dideuterohydroxymethyl)-2,2,5,5-tetrakis(trideuteromethyl)-2,5-dihydropyrrol-1-oxyl

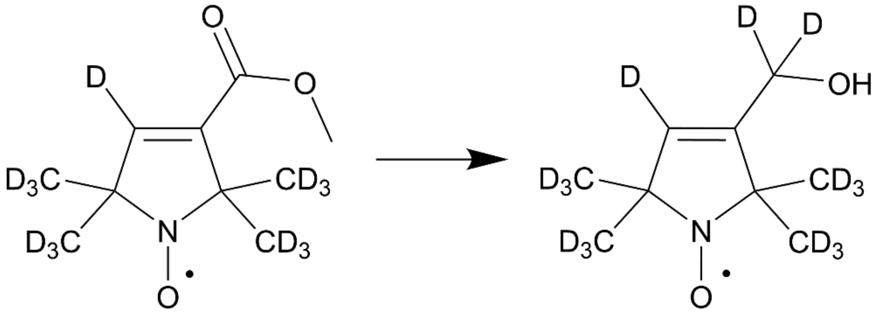

$C_{10}H_3D_{13}NO_3^{\bullet}$ M = 211.32 g/mol

$C_9HD_{15}NO_2^{\bullet}$ M = 185.32 g/mol

Methyl (4-deutero-1-oxyl-2,2,5,5-tetrakis(trideuteromethyl)-2,5-dihydropyrrole-3-)carboxylate[1] (97.9 mg, 463 µmol, 1.0 equivalents) was dissolved in anhydrous and degassed tetrahydrofuran (4 mL) under inert atmosphere at 0 °C. $LiAlD_4$ (25.0 mg, 596 µmol, 1.3 equivalents) was slowly added. The solution was allowed to warm to room temperature and stirred for 48 h. Solids were removed by filtration and the filtrate was extracted with ethyl acetate (6 × 15 mL). The combined organic phases were dried over $MgSO_4$. The solvent was removed under reduced pressure and the residue was purified by column chromatography (hexane / ethyl acetate (1:1)). The solvent was removed from the combined fractions and the residue crystallized (DCM / Hexane (1:1)) to result in yellow crystals.

[1] Methyl (4-deutero-1-oxyl-2,2,5,5-tetrakis(trideuteromethyl)-2,5-dihydropyrrole-3-)carboxylate *was available from a previous project, publication in preparation*

Synthesis of (4-deutero-1-oxyl-2,2,5,5-tetrakis(trideuteromethyl)-2,5-dihydropyrrol-3-yl)dideuteromethyl methanesulfonate

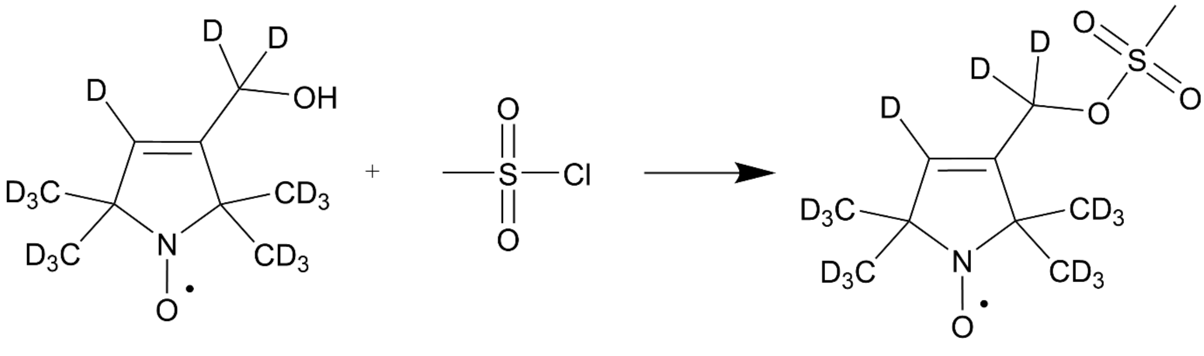

$C_9HD_{15}NO_2^{\bullet}$
M = 185.32 g/mol

CH_3ClO_2S
M = 114.54 g/mol

$C_{10}H_3D_{15}NO_4S^{\bullet}$
M = 263.41 g/mol

4-Deutero-3-(dideuterohydroxymethyl)-2,2,5,5-tetrakis(trideuteromethyl)-2,5-dihydropyrrol-1-oxyl (8.87 mg, 48 µmol, 1.0 equivalents) was dissolved in dry CH_2Cl_2 under inert atmosphere. The solution was cooled to 0 °C and triethylamine (7.35 µL, 53 µmol, 1.1 equivalents) was added. Methane sulfonyl chloride (4.08 µL, 53 µmol, 1.1 equivalents) was added dropwise and the reaction was stirred for 3 h at room temperature. CH_2Cl_2 was added and the solution was washed with $NaHCO_3$ solution (5% [w/v] in H_2O, 30 mL) and H_2O (30 mL) before drying the organic phase over Na_2SO_4. The solvent was removed to obtain the product as yellow crystals.

Synthesis of 3-iodomethyl-2,2,5,5-tetramethyl-2,5-dihydropyrrol-1-oxyl/4-deutero-3-(dideuteroiodomethyl)-2,2,5,5-tetrakis(trideuteromethyl)-2,5-dihydropyrrol-1-oxyl

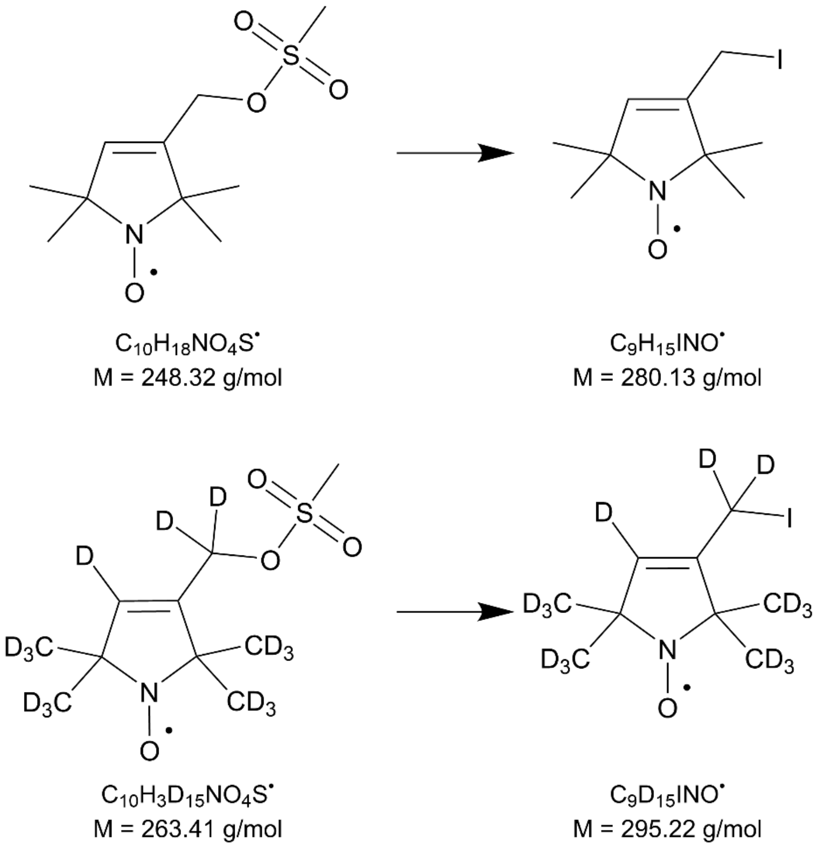

3-Iodomethyl-2,2,5,5-tetramethyl-2,5-dihydropyrrol-1-oxyl and 4-deutero-3-(dideuteroiodomethyl)-2,2,5,5-tetrakis(trideuteromethyl)-2,5-dihydropyrrol-1-oxyl, used for the spin labelling of the RNA, were prepared according to Qin *et al.*[11] Briefly, (1-oxyl-2,2,5,5-tetramethyl-2,5-dihydropyrrol-3-yl)methyl methanesulfonate (Toronto Research Chemicals, Toronto, Canada) or it's deuterated equivalent (4-deutero-1-oxyl-2,2,5,5-tetrakis(trideuteromethyl)-2,5-dihydropyrrol-3-yl)dideuteromethyl methanesulfonate (see above) (1 mg, 4.0 µmol/3.8 µmol) was dissolved in acetone (200 µL) and combined with sodium iodide in acetone (1 M, 3 µL equivalent to 450 µg, 3.0 µmol, 0.75 equivalents). The mixture was incubated at 37 °C for 1 h. During incubation a white precipitate formed. The solution was centrifuged for 10 min at 13000 × *g* and the supernatant was recovered. The precipitate was washed with acetone, centrifuged, and the supernatant was recovered. The supernatants were combined and the solvent was removed using a SpeedVac™ vacuum concentrator.

1.6. Labelling and purification of the fluoride riboswitch

The fluoride riboswitch RNA constructs with a phosphorothioate modification in either positions C14/C44, G29/G36, A9/A49, C4/C17, G5, G43, or U46 were purchased High-performance liquid chromatography (HPLC) purified and freeze-dried (Integrated DNA Technologies, Leuven, Belgium) and dissolved to a concentration of 500 µM. The spin labelling was performed according to the protocol of Qin *et al*.[11] Briefly, the iodinated spin label was dissolved in acetonitrile. Buffer (50 mM KOAc pH 6.1, 5 mM $Mg(OAc)_2$, 500 µM KF), RNA (12.5 µL), and DEPC treated MilliQ® water as required were added to result in a reaction mixture with a volume of 20 µL. The reaction mixture was incubated in the dark at room temperature for 12-24 h.

The purification of the RNA from excess spin label was performed using the Monarch® RNA Cleanup kit (New England Biolabs, Ipswich, United States). The RNA was eluted with DEPC treated H_2O or D_2O as required. The labelled RNA was freeze-dried if needed and re-dissolved in H_2O or D_2O as required.

1.7. Concentration determination

The concentration of the RNA was determined by UV/vis spectroscopy using a nanophotometer. The absorption of the RNA was determined at a wavelength of 260 nm and the concentration calculated according to the Lambert-Beer-Law. For the analysis 1-2 µL of a dilute RNA solution were applied. The absorption of the solvent was determined using deionised water or D_2O as applicable and subtracted from the spectrum of the RNA.

1.8. Sample preparation for spectroscopy

Nuclear magnetic resonance (NMR) samples:

The RNA was folded according to the folding protocol, $Mg(OAc)_2$ (up to 5 mM) and KF (500 µM) were added as required. 10% D_2O were added to the sample. The sample was transferred to a DEPC treated Shigemi tube (5 mm, BMS-005, D_2O matched, Merck, Glasgow, United Kingdom) with a sample volume of 250 µL.

Continuous wave (CW) electron paramagnetic resonance (EPR) samples:

The concentration of the RNA was adjusted to 12.5 - 20 µM. Measurements were performed in DEPC treated 20 µL or 10 µL capillaries (Blaubrand® intraMARK micropipettes, Brand, Wertheim, Germany) with a sample volume of 20 µL or 10 µL, respectively. The capillaries were sealed with wax (Vitrex, Herlev, Denmark) or critoseal® (Fisher Scientific, Schwerte, Germany).

Q-band EPR samples:

PELDOR samples: The RNA in deuterated buffer was folded according to the folding protocol. For analysis of the free state no further ions were added, for the *apo* form $Mg(OAc)_2$ (5 mM), and for the *holo* form $Mg(OAc)_2$ (5 mM) and KF (500 µM) were added. 20% (v/v) glycerol-d_8 was added as cryoprotectant to all samples. The RNA concentration was adjusted to 10 µM (C14/C44, G29/G36, A9/A49) or 20 µM (C4/C17). Measurements were performed in DEPC treated 3 mm quartz tubes with a sample volume of 65 µL. The sample was transferred into the tube and flash frozen in liquid nitrogen.

^{19}F ENDOR samples: The RNA in deuterated buffer was folded according to the folding protocol. $Mg(OAc)_2$ (5 mM) and KF (500 µM) were added if not mentioned otherwise and 20% (v/v) glycerol-d_8 was added as cryoprotectant. The RNA concentration was adjusted to 235 µM. Measurements were performed in DEPC treated quartz capillaries (Wilmad Q-band CFQ EPR tube, both ends open, 100 mm length, 1.6 mm O.D.) with a sample volume of 12 µL. The sample was transferred into the tube and flash frozen in liquid nitrogen.

W-band EPR samples:

^{19}F ENDOR samples: The RNA in deuterated buffer was folded according to the folding protocol. $Mg(OAc)_2$ (5 mM) and KF (500 µM) were added if not mentioned otherwise and 20% (v/v) glycerol-d_8 was added as cryoprotectant. The RNA concentration was adjusted to 100-150 µM. Measurements were performed in DEPC treated quartz capillaries (Wilmad W-band suprasil EPR tube, both ends open, 40 mm length, 0.9 mm O.D.) with a sample volume of 2 µL. The sample was transferred into the tube and flash frozen in liquid nitrogen.

1.9. NMR measurements

NMR measurements were performed at a Bruker AVIII-HD 700 spectrometer (Bruker UK Limited, Coventry, United Kingdom) equipped with a CryoProbe Prodigy TCI probe head. The temperature was set to 295 K if not mentioned otherwise. All spectra were recorded with the pulse sequence zgesgp[12] at a centre field of 4.69 parts per million (ppm), a spectral width of 20 ppm, and 1024 scans.

The spectra were analysed using TopSpin 3.6 pl1 (Bruker BioSpin). The phase was corrected and the line broadening was set to 2.0.

1.10. EPR measurements and data analysis

1.10.1. PELDOR measurements

PELDOR measurements were performed at an ELEXYS E580 EPR spectrometer (University of St Andrews) from Bruker operating at Q-band (34 GHz) microwave frequency and equipped with a 3 mm cylindrical resonator (QT-II) and a cryogen-free variable temperature cryostat (Cryogenic Ltd, London, United Kingdom). Pulses were amplified by a traveling wave tube amplifier (150 W) (Applied Systems Engineering, United States).

For PELDOR measurements[13] were performed with the field position corresponding to the maximum of the field swept spectrum of the nitroxide. A frequency-offset (pump – detection frequency difference) of +80 MHz and a shot repetition time (SRT) of 3 ms were used. τ_1 was set to 380 ns and τ_2 was chosen as 3 µs. Pulse lengths were set to 16 ns and 32 ns for $\pi/2$ and π, respectively, and optimised on maximum signal. Measurements were performed using an arbitrary waveform generator with a 16 step phase cycle.[14] The pump pulse was set to the maximum of the spectrum and resonance frequency of the resonator.

PELDOR data was analysed using the MATLAB (MathWorks, Natick, MA, United States) program DeerAnalysis2022.[15,16] The raw experimental data were background corrected and a Tikhonov regularization was performed. The optimal regularization parameter was set as suggested by the software and a validation with 16 steps in the range of 5% – 80% of the dipolar evolution time was performed. The optimised background start value from this validation was used in a second Tikhonov regularization. A second validation was performed with the optimised regularization parameter and with white noise added with a level of 1.50 and 50 trials. Data with a root mean square deviation (RMSD) within 15% of the lowest value were kept. In case the slope of the calculated background function was positive, which corresponds to a negative concentration and is therefore physically not possible, the experimental time window was shortened by 10% and the evaluation was repeated. The resulting distance distribution and the corresponding colour bars were obtained. The colour bar is showing the reliability ranges of the distance distribution (green: shape reliable, yellow: mean and width reliable, orange: mean reliable, red: no quantification possible).

For data analysis with DeerNet[17] raw time traces were processed using DeerNet in Spinach 2.8.6280. Data were pre-processed with the "elexsys2deernet" function and DeerNet was executed with standard parameters specifying the "deer" experiment. Traces and distance distributions were extracted and plotted.

Data analysis with DeerLab[18] was performed in Python 3.9 using the script for basic analysis of a 4-pulse DEER signal. Data was loaded, the parameters were set according to the measurement and the

bootstrapping was performed with 250 iterations. The resulting fit of the time trace with the unmodulated contribution as well as the distance distribution calculated in steps of 0.5 Å with the confidence intervals were extracted and plotted.

1.10.2. ^{19}F ENDOR measurements

^{19}F ENDOR measurements at Q-band (34 GHz) microwave (MW) frequency were performed on a Bruker ELEXYS E580 EPR spectrometer (MPI Göttingen) equipped with an ENDOR resonator (EN65107D2) inserted into a helium gas flow cryostat (Oxford Instruments, Abingdon, United Kingdom). A 170 W traveling wave tube amplifier (Applied systems engineering, United States) was used. The pulse length was optimised resulting in a typical π/2 pulse in the range of 10-12 ns. Measurements were performed at a temperature of 50 K. RF pulses were amplified using a 250 W RF amplifier (250A250A, Amplifier Research) or a 600 W RF amplifier (600A225A, Amplifier Research) and the pulse length was set to 50 µs or 200 µs. SRTs for ENDOR measurements were set in the order of 6-9 ms. The τ-value was optimised to 2600 ns and 4000 ns for G5 and U46, respectively. For the acquisition stochastic RF acquisition with 10 shots per point (SPP) and 1 SPP was used for G5 and U46, respectively. The integrator gate width was set to 20 ns and 48 ns for G5 and U46, respectively, and placed symmetrically around the maximum of the echo.

^{19}F ENDOR measurements at W-band (94 GHz) MW frequency were performed on a Bruker ELEXYS E680 EPR spectrometer (MPI Göttingen) equipped with a Bruker ENDOR cylindrical resonator (EN600-1021H) inserted into a helium gas flow cryostat (Oxford Instruments). A 2 W amplifier was used. The pulse length was optimised resulting in a typical π/2 pulse of 10 ns. Measurements were performed at a temperature of 50 K. Radio frequency (RF) pulses were amplified using a 250 W RF amplifier (250A250A, Amplifier Research) and the pulse length was set to 50 µs. SRTs for ENDOR measurements were set in the order of 3-7 ms. The τ-value was optimised to 3000 ns and 3200 ns for G5 and G43, respectively. Stochastic RF acquisition with 10 SPP was used. The integrator gate width was set to 20 ns, placed symmetrically around the maximum of the echo.

The ^{19}F ENDOR spectra were normalised by number of scans and video gain, baseline corrected, shifted to $\nu_{RF} - \nu(^{19}F)$ along the x-axis, and summed up to result in the sum spectrum.

^{19}F ENDOR data was analysed using the software SimSpec.[19] SimSpec is a fast simulation routine using the diagonalisation of the spin Hamiltonian to predict the spectrum. Since it is a static simulation routine it allows for calculation of many spectra (here up to 751 spectra for different coupling constants, orientations, or a combination of those) within a few minutes (up to ~5 minutes). The simulation of Mims ENDOR spectra including the Mims blind spot function $p_{seq,k}^{Mims} = 1 - \cos(2\pi A_k \tau)$

is included in SimSpec. All simulations were performed using the 'Sophe' grid with 200 knots and Lorentzian line shape for the convolution of the RF resonances. The ENDOR linewidth parameter was set to 14 kHz for G5 and G43 and 7 kHz for U46. The experiment parameters were set to 'Mims'. Lengths of the MW and RF pulses for the simulation were set according to the experimental parameters.

For simulations a Gaussian distance distribution with the mean distance according to the read-off coupling constant from the ^{19}FENDOR sum spectrum was calculated in steps of 0.2 Å. The full width at half maximum (FWHM) of the Gaussian distance distribution used for simulations was optimised to result in a well matching simulation of the measured spectrum. The whole Gaussian distance distribution could be well described with 401-751 points. Euler Angles were set to a single orientation. The ENDOR spectra were simulated as described above and weighted by the intensity of the Gaussian distribution at the respective distance. The spectrum simulated in this way can only be applied for the sum spectrum, since orientation selection was neglected in this approach.

For simulations accounting for the label orientations the Euler angles and distances were taken from the rotamers from *in silico* spin labelling. A spectrum was simulated for each rotamer. In case of MtsslSuite all spectra were summed to form the ensemble spectrum. In case of MMM spectra were either summed to the ensemble spectrum or multiplied by the population factor and summed to form the weighted sum spectrum.

2. NMR characterisation of unlabelled samples

2.1. ^{1}H NMR analysis at room temperature

Using our folding protocol the imino region (11-14.5 ppm) of the ^{1}H NMR spectrum shows the same signals for the free and *holo* forms (Figure S1 (a) and (c), respectively) of the riboswitch as previously published by Ren *et al.*[20] Changes are traced based on four characteristic signals at 11.75 ppm, 12.2 ppm, 13.9 ppm, and 14 ppm, highlighted by vertical lines in the spectra.

Starting from the free form, characterised by sharp resonances at 14 ppm and 12.2 ppm (Figure S1 (a)), addition of $Mg(OAc)_2$ leads to a decrease in intensity of those two signals and slightly sharper signals between 12.5 ppm and 13.5 ppm (Figure S1 (b)). Subsequent addition of KF, resulting in the *holo* form, leads to disappearance of the signals at 12.2 ppm and 14 ppm while characteristic signals at 11.75 ppm and 13.9 ppm arise (Figure S1 (c)). This change of the signals in the imino region of the ^{1}H NMR leads to the conclusion that a Mg^{2+}-bound *apo* form seems to be present when only adding Mg^{2+} to the free form (Figure S1 (b)) as the *apo* form exhibits different resonances for the base pairs compared to *free* and *holo* aptamers. In general, for all three forms described here signals can be seen in the ^{1}H NMR spectrum. Those signals evidence base pairing throughout all three forms, indicating presence of tertiary structure of the riboswitch in all three forms.

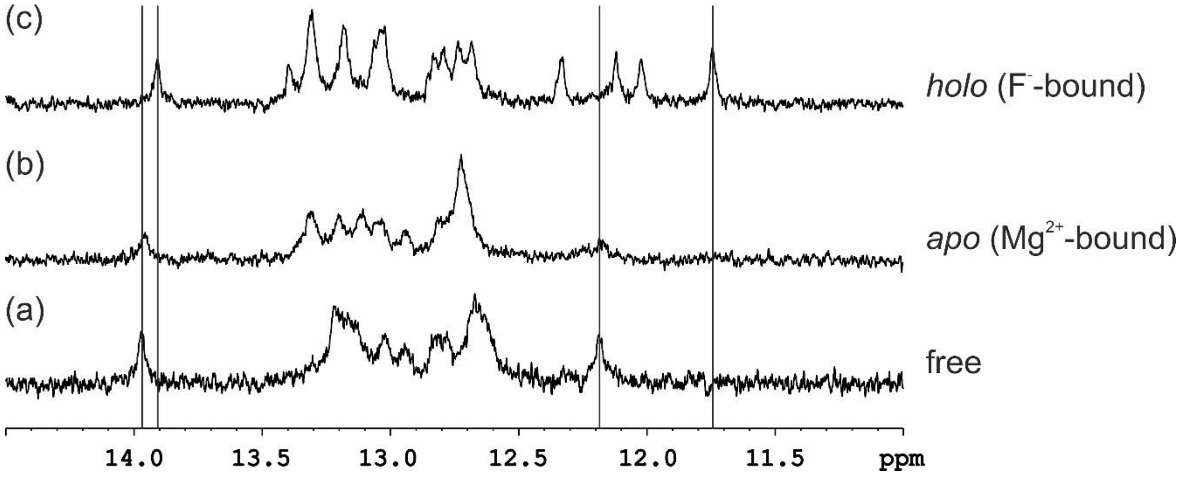

Figure S1: Imino region of the ^{1}H NMR of the free (a), Mg^{2+}-bound apo (b), and F^--bound holo (c) forms of the fluoride riboswitch measured at 700 MHz and 295 K. Buffer conditions: 25 µM RNA, 50 mM KOAc pH 6.1, 10% D_2O (a), 25 µM RNA, 50 mM KOAc pH 6.1, 5 mM $Mg(OAc)_2$, 10% D_2O (b), 25 µM RNA, 50 mM KOAc pH 6.1, 5 mM $Mg(OAc)_2$, 500 µM KF, 10% D_2O (c).

The influence of Mg^{2+} on the prevalent form of the fluoride riboswitch has been further investigated by a titration series, increasing the amount of Mg^{2+}, with the riboswitch in presence of F^- (Figure S2). Spectra show a gradual increase of the signals at 11.75 ppm and 13.9 ppm while the signals at 12.2 ppm and 14 ppm gradually decrease.

This indicated the formation of the F^--bound form upon addition of Mg^{2+}. From this we concluded it depends on the concentration of Mg^{2+}. This is also in agreement with the finding that the K_D for F^-

binding is higher at 1 mM $Mg(OAc)_2$ compared to 5 mM $Mg(OAc)_2$.[20] Therefore, sufficiently high Mg^{2+} concentrations are needed for the formation of the Mg^{2+}-bound form capable of forming the coordinative Mg^{2+} triangle capable of binding F^- ions.

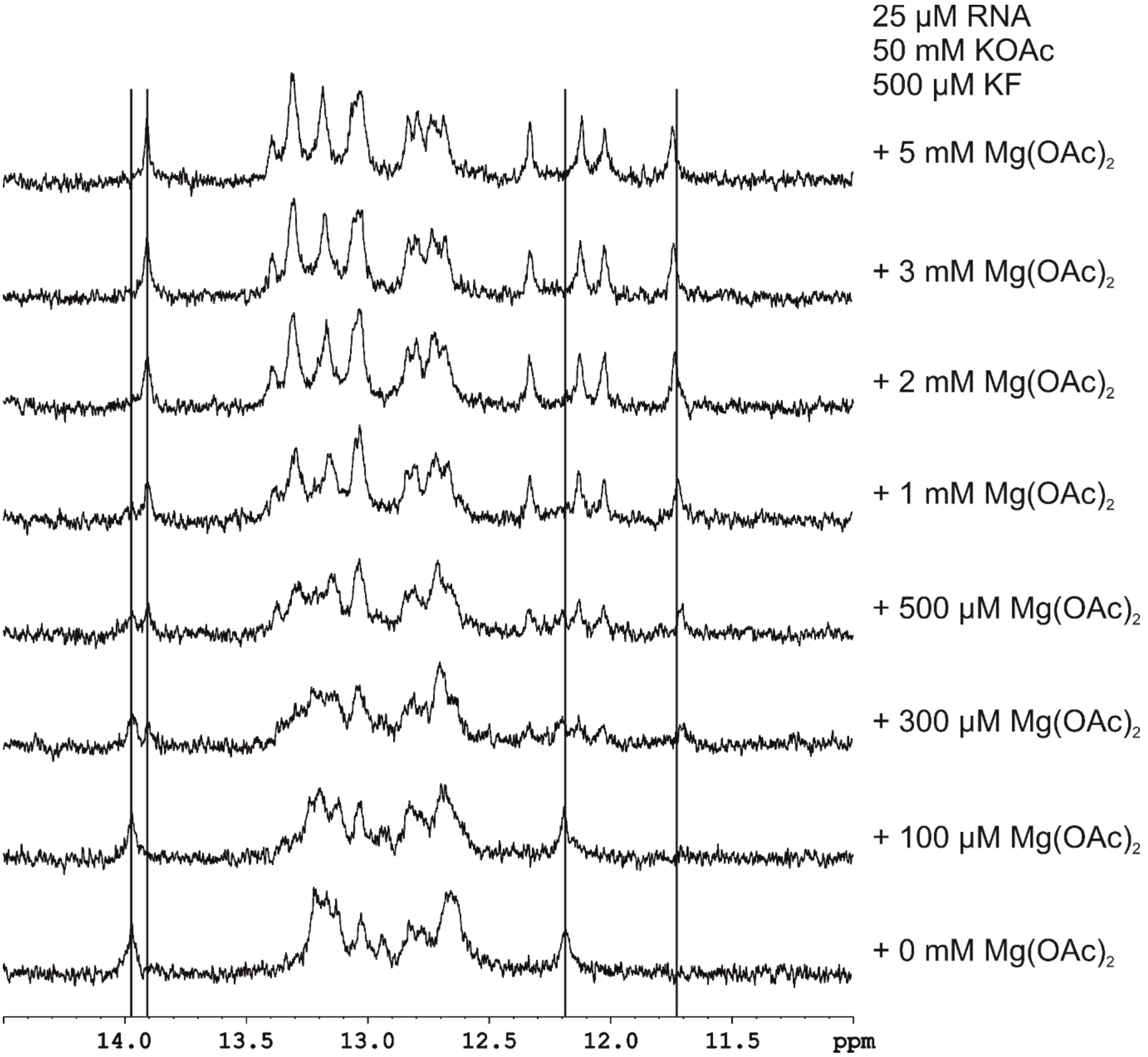

Figure S2: Imino region of the ^1H NMR of a titration series of the fluoride riboswitch with increasing amount of Mg^{2+} in presence of F^- measured at 700 MHz and 295 K. Buffer conditions: 25 µM RNA, 50 mM KOAc pH 6.1, 500 µM KF, $Mg(OAc)_2$ as indicated in the figure, 10% D_2O.

2.2. ^{1}H NMR analysis at decreased and elevated temperatures

To ensure that the riboswitch does not undergo a transition into a more structured, energetically favoured form due to freezing of PELDOR and ENDOR samples the folding was also analysed at 4 °C (Figure S3). The imino region of the ^{1}H NMR spectra does not show a higher organisation of the *free* and the *apo* forms at the decreased temperature compared to the room temperature measurements, indicating that decreased temperatures do not lead to a more structured form.

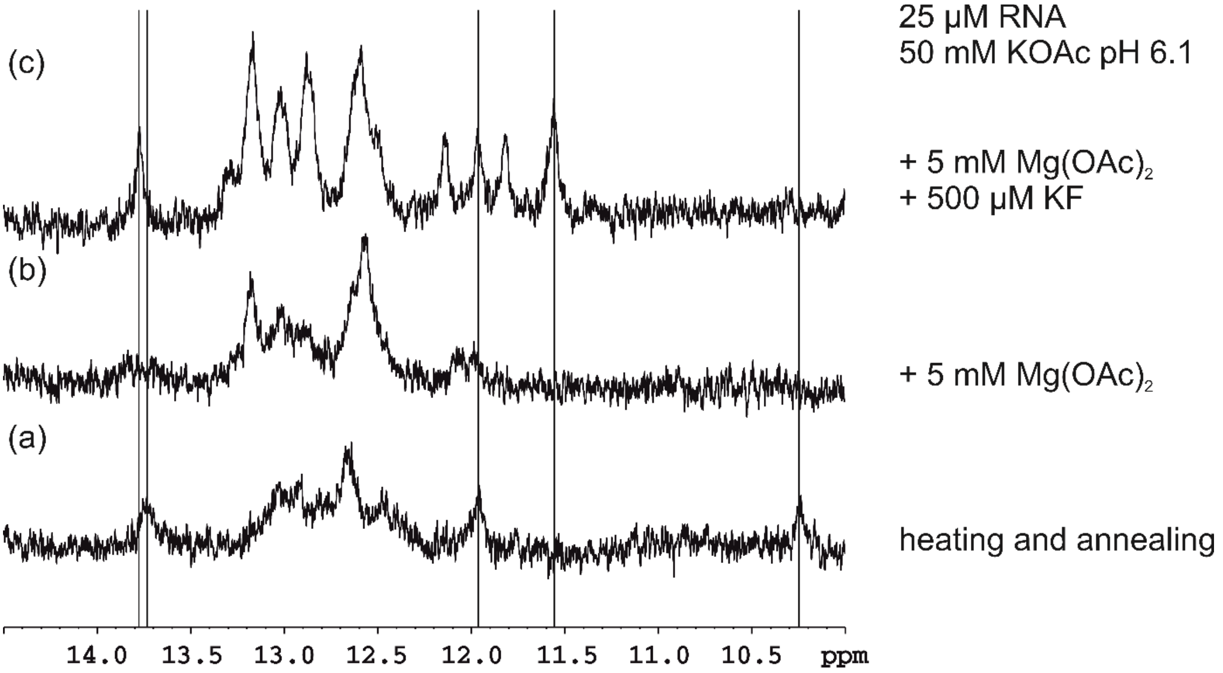

Figure S3: Imino region of the ^{1}H NMR of the free (a), apo (b), and holo (c) form of the fluoride riboswitch measured at 700 MHz and 277 K. Buffer conditions: 25 µM RNA, 50 mM KOAc pH 6.1, 10% D_2O (a), 25 µM RNA, 50 mM KOAc pH 6.1, 5 mM $Mg(OAc)_2$, 10% D_2O (b), 25 µM RNA, 50 mM KOAc pH 6.1, 5 mM $Mg(OAc)_2$, 500 µM KF, 10% D_2O (c).

Since *T. petrophila* is a thermophilic bacterium the stability of the tertiary structure was also investigated at an elevated temperature of 60 °C (Figure S4). The imino region of the ^{1}H NMR spectrum is still showing signals originating from base pairs. Compared to the room temperature spectrum the signals are generally shifted towards higher ppm values. In addition, not each signal is shifted to the same extent. This difference is due to the dynamics of the residues at higher temperature. Nevertheless, the spectra exhibit high similarity showing that the riboswitch is structured at 60 °C.

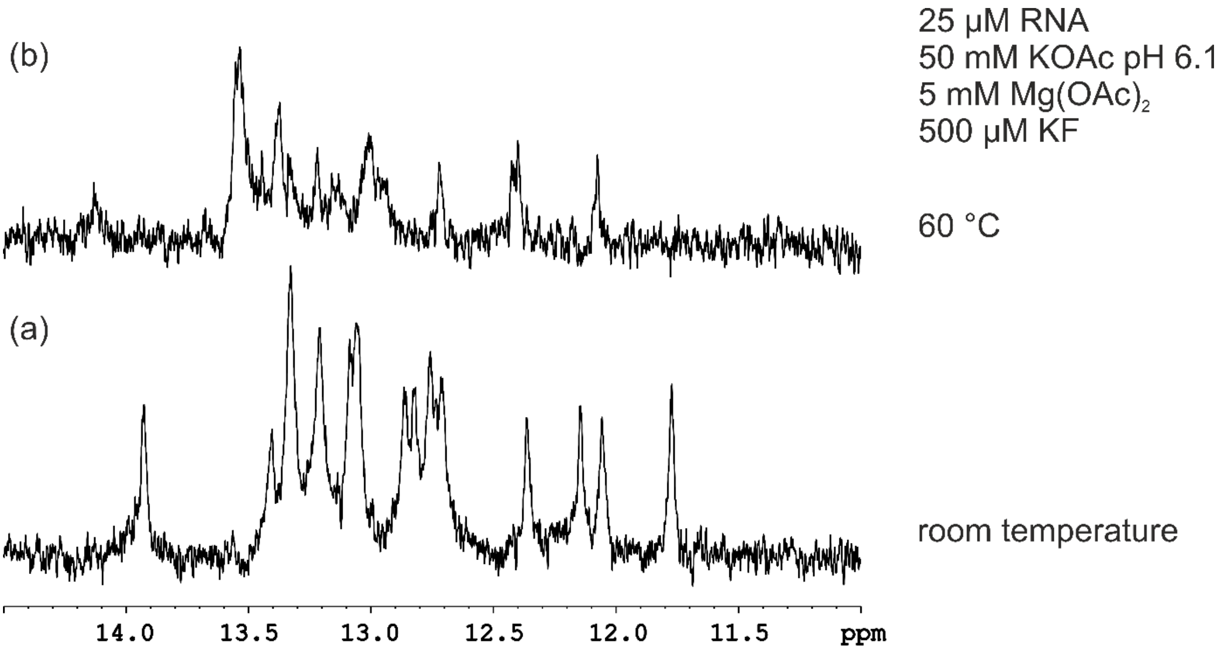

Figure S4: Imino region of the ^{1}H NMR of the holo form of the fluoride riboswitch measured at 700 MHz and 295 K (a) and 333 K (b). Buffer conditions: 25 µM RNA, 50 mM KOAc pH 6.1, 5 mM $Mg(OAc)_2$, 500 µM KF, 10% D_2O.

3. Selection of labelling sites

3.1. Selection of labelling sites for PELDOR measurements

For the selection of suitable labelling sites for PELDOR experiments a site scan was performed with MMM. All pairs in a distance range 1.8 – 4.5 nm were further considered. To obtain a well-defined distance distribution with a single mean distance and a sharp peak the output was sorted by RMSD of the N-O midpoint coordinate between the rotamers and the first 50 combinations were further analysed. For these combinations the relative width of the distance distribution (relative width = standard deviation/mean distance) was below 15% throughout, also indicating a sharp distance distribution. Combinations including the residues involved in Mg^{2+} coordination (A6, U7, G8, U41, G42) were excluded and the remaining combinations were sorted (Table S2, Figure S5) into 4 groups, also considering neighbouring residues and the most frequently appearing residues. One pair of each group was selected, ensuring that we could probe preorganisation and a change in the pseudoknot formation between the free, *apo* and *holo* forms. The combinations C14/C44, G29/G36, A9/A49, and C4/C17 were chosen for labelling. The distance distributions (Figure S6) derived from *in silico* labelling from MtsslSuite (blue) and MMM (green) are of similar mean distance and width.

Table S2: Groups A-D formed from the possible combinations of the most frequently appearing labelling sites. Colour descriptions of the groups are referring to the graphical representation in Figure S5.

Group	Residue 1	Residue 2
A (red)	G3, C4 or G5	C17, C18, A19 or A20
B (green)	C14, G15, C16 or C17	G43, C44 or C45
C (blue)	U28 or G29	G36 or C37
D (magenta)	A9 or G10	A49 with C50

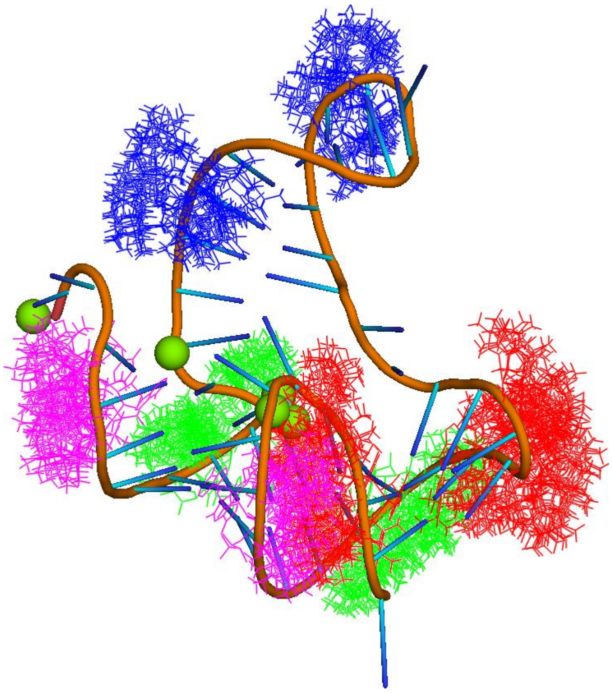

Figure S5: Graphical representation of the fluoride riboswitch (4ENC) with the possible spin label conformations for group A (red), B (green), C (blue), and D (magenta) as described in Table S2. Spin labels were obtained from MMM.

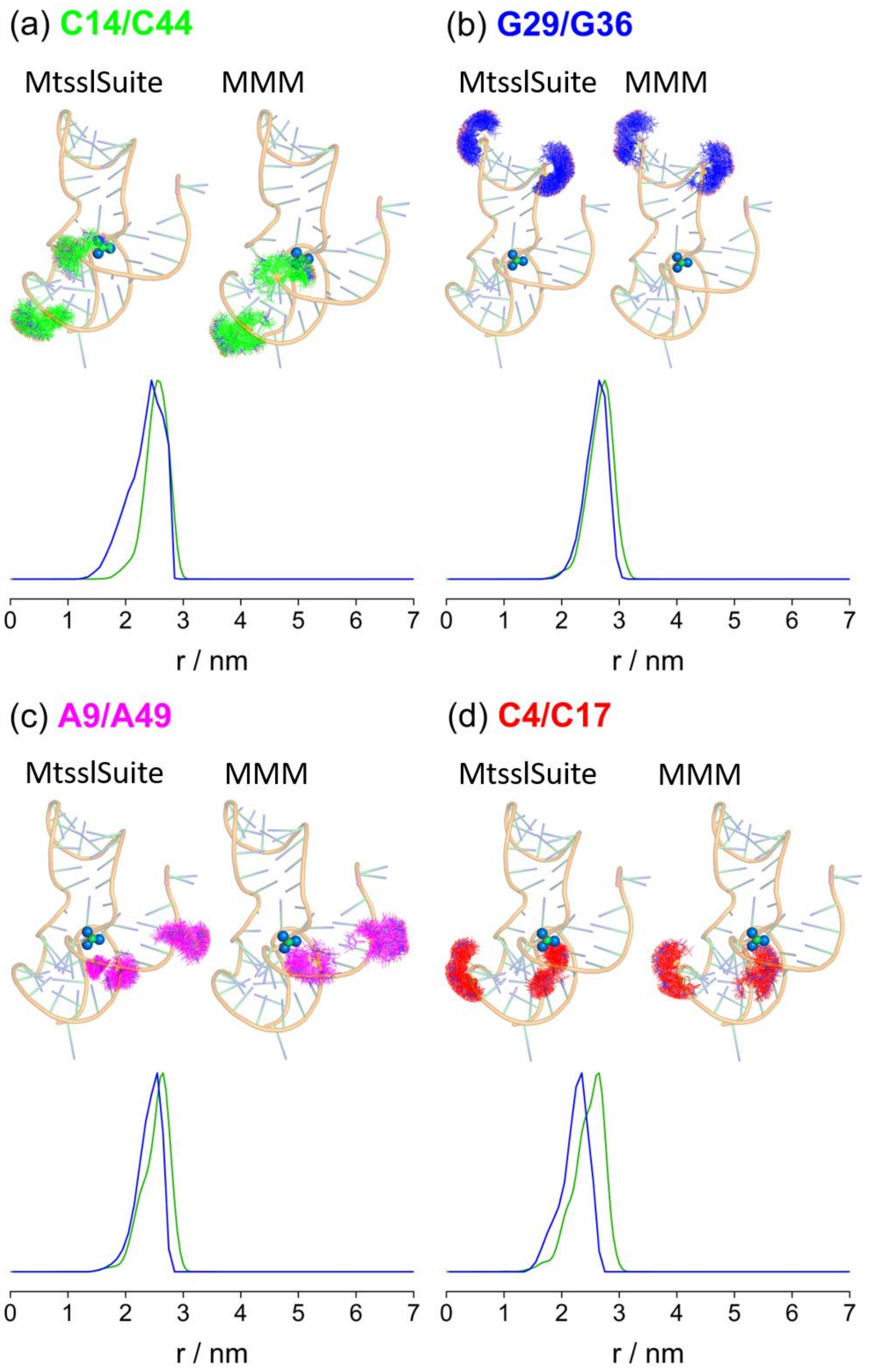

Figure S6: Graphical representation of the rotamer distributions obtained from MtsslSuite and MMM (programme as indicated in the figure) attached to the crystal structure of the fluoride riboswitch (4ENC) with the F^- (green sphere) and the Mg^{2+} (blue spheres) as well as the respective distance distributions between the spin labels of the three different spin label pair combinations C14/C44 (a), G29/G36 (b), A9/A49 (c), and C4/C17 (d) from in silico spin labelling with MtsslSuite (blue) and MMM (green).

3.2. Selection of labelling sites for ^{19}F ENDOR measurements

For the initial selection of the labelling sites MMM has been used. All residues have been spin labelled *in silico* and the distance distribution towards the F^- has been calculated. Residues involved in the coordination of Mg^{2+} ions were removed from the list of potential labelling sites. In addition, all residues showing either too long or not well resolved (broadened distance distributions or distance distributions with a splitting not well resolved) were removed from possible labelling sites. In the further progress of the project the spin label used by us has also been implemented into MtsslSuite (blue line) allowing us to show the distance distributions from both programmes here.

We decided to take G43 as one of the labelling positions since it is in a central position of all remaining possible sites, either C4 or G5, and either C44 or C45 or U46, resulting in 6 potential combinations. The most suitable positions were selected based on the formation of a preferably wide-spread triangle around the F^- to have three linearly independent distance vectors between the respective spin label and the F^- (Figure S7). This could be seen for the combination G5-G43-U46 (Figure S7 (f)).

The *in silico* spin labelling with MtsslSuite shows a well-defined distance distribution with only a single mean distance and a FWHM around 2 Å for each of the two constructs G5 and G43. The distance distributions from MMM show a shoulder at lower distance for both constructs (Figure S8). For U46 two distance contributions are predicted by both, MtsslSuite and MMM. The shorter distance is at about 15 Å with the mean distance predicted by MMM being about 2 Å shorter than the one predicted by MtsslSuite. The longer distance contribution is above the resolvable distance limit of 20 Å.

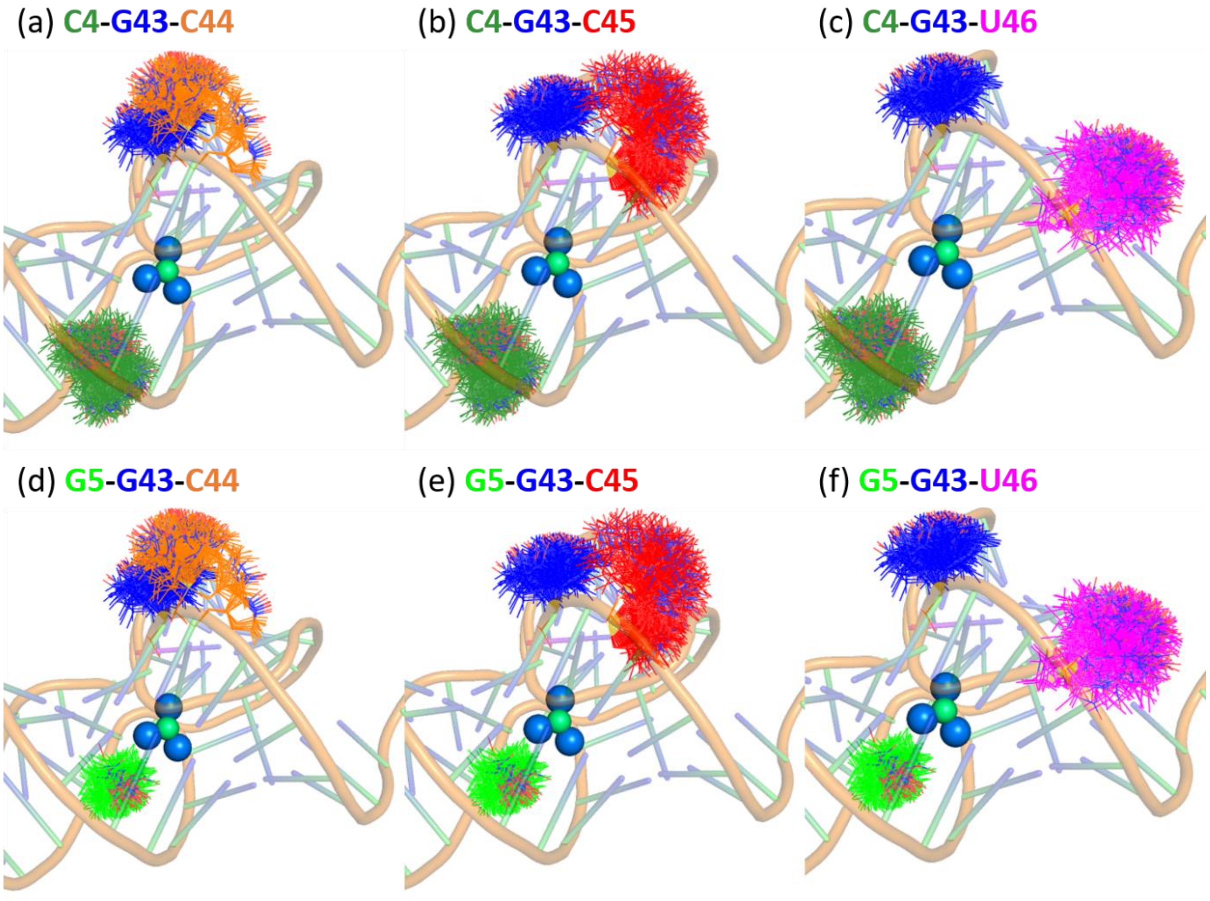

Figure S7: Graphical representation of the potential spatial orientations of the spin labels around the F^- (green sphere with Mg^{2+} represented as blue spheres). Spin labels are obtained from MtsslSuite. Combinations were built from C4 (dark green, upper row) or G5 (light green, bottom row) with G43 (blue) and C44 (orange, left column) or C45 (red, middle column) or U46 (magenta, right column).

A comparison of the graphical representations of the rotamer clouds for the selected residues from MtsslSuite and MMM as well as the corresponding distance distributions are given in Figure S8. The cartoons demonstrate that in general the rotamers from MMM show a wider spatial spread compared to the ones from MtsslSuite. This is also represented in the broader distance distributions from MMM compared to MtsslSuite.

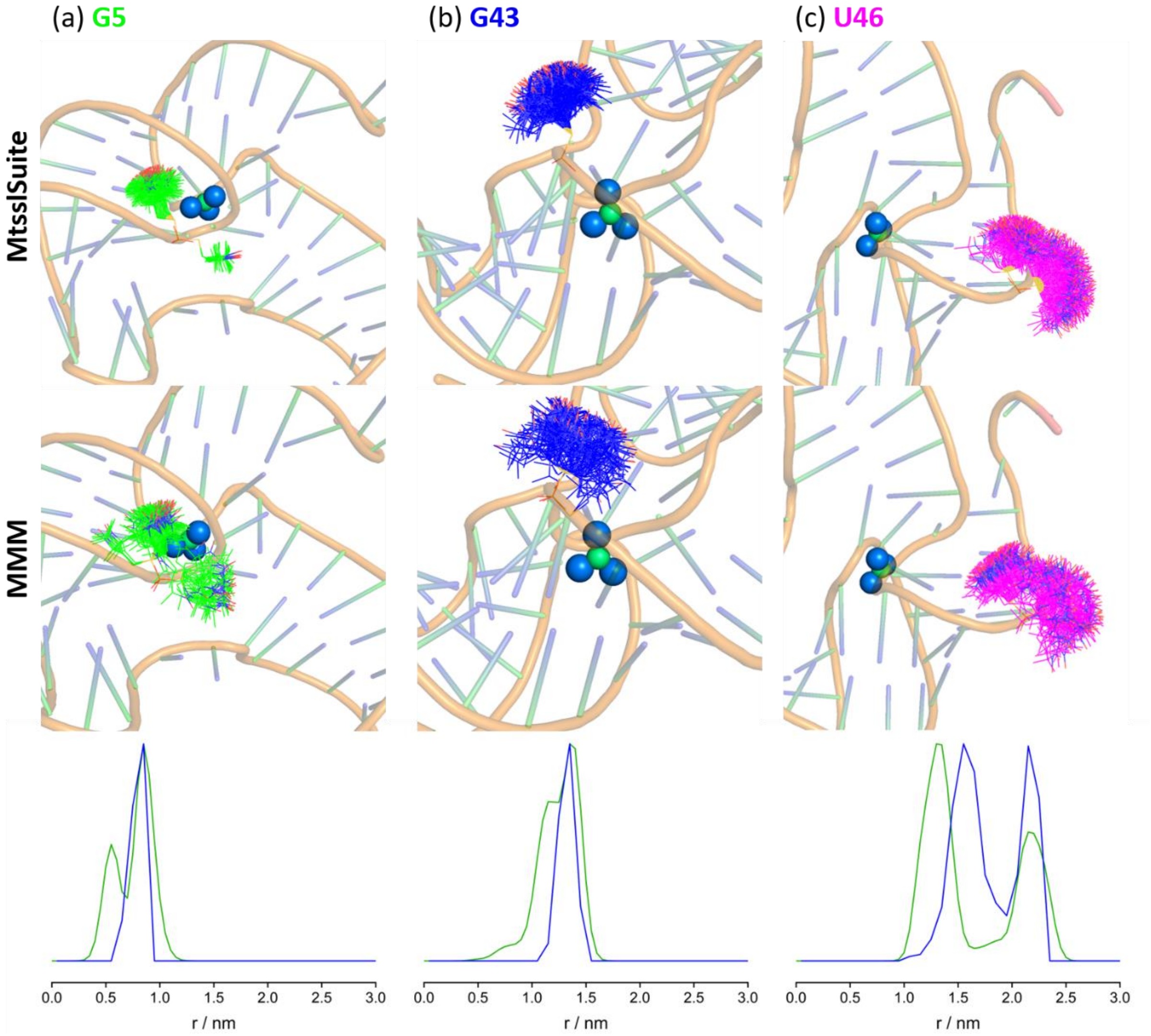

Figure S8: Graphical representation of the rotamer clouds obtained from MtsslSuite (top row) and MMM (middle row) attached to the crystal structure of the fluoride riboswitch with the F^- (green sphere) and the Mg^{2+} (blue spheres) as well as the respective distance distributions (bottom row) between the spin label and the F^- from MtsslSuite (blue) and MMM (green) for the three different constructs G5 (a), G43 (b), and U46 (c) used in the ^{19}F ENDOR investigation.

3.3. Identification of diastereomers in *in silico* labelling for ^{19}F ENDOR measurements

Whereas the spin label from the *in silico* spin labelling with MMM accounts for both diastereomeric labelling positions by default, those had to be separately introduced in MtsslSuite and were separately evaluated for further processing of the spectra. Respective distance distributions are shown in Figure S9. The evaluation of the rotamer ensembles resulted in the number of rotamers for the different labelling positions as given in Table S3.

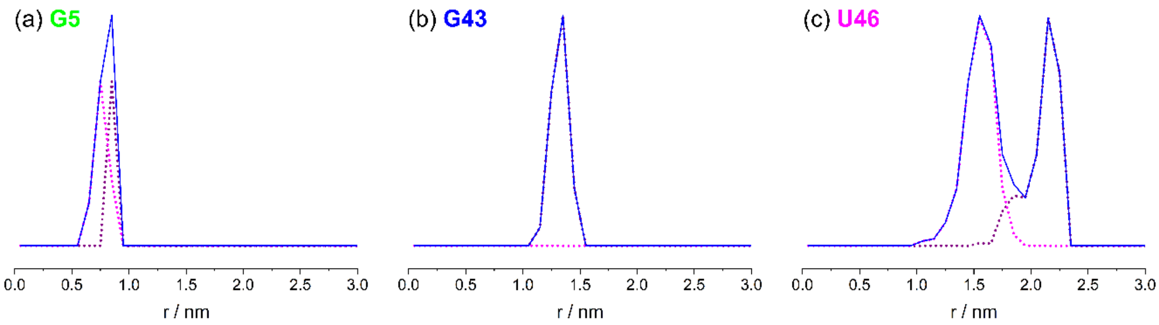

Figure S9: Distance distributions for the RNA constructs designed for ^{19}F ENDOR measurements from MtsslSuite in blue with dotted distributions representing the separate diastereomers (diastereomer 1 in purple, diastereomer 2 in magenta).

Table S3: Rotamers extracted from rotamer clouds from in silico spin labelling.

Construct	MMM	MtsslSuite	
		Diastereomer 1	Diastereomer 2
G5	108	13	200
G43	102	200	0
U46	241	200	200

4. Analysis of the spin-labelled fluoride riboswitch constructs

The selected fluoride riboswitch constructs were labelled and purified. The labelling efficiency was determined by a CW EPR spin count to be 51% to 82%.

To ensure that the introduction of the spin label does not interfere with the folding of the fluoride riboswitch, comparison of the ^{1}H NMR spectra in the free and *holo* forms have been performed for all labelled constructs (Figure S10 to Figure S16).

A structural change from the *free* to the *holo* form can be observed for all constructs showing that the folding of the riboswitch is not compromised by the attached label. Splitting of the signals in the ^{1}H NMR spectrum can be traced back to the chiral centre introduced by the phosphorothioate modification and the spin label in the respective position.

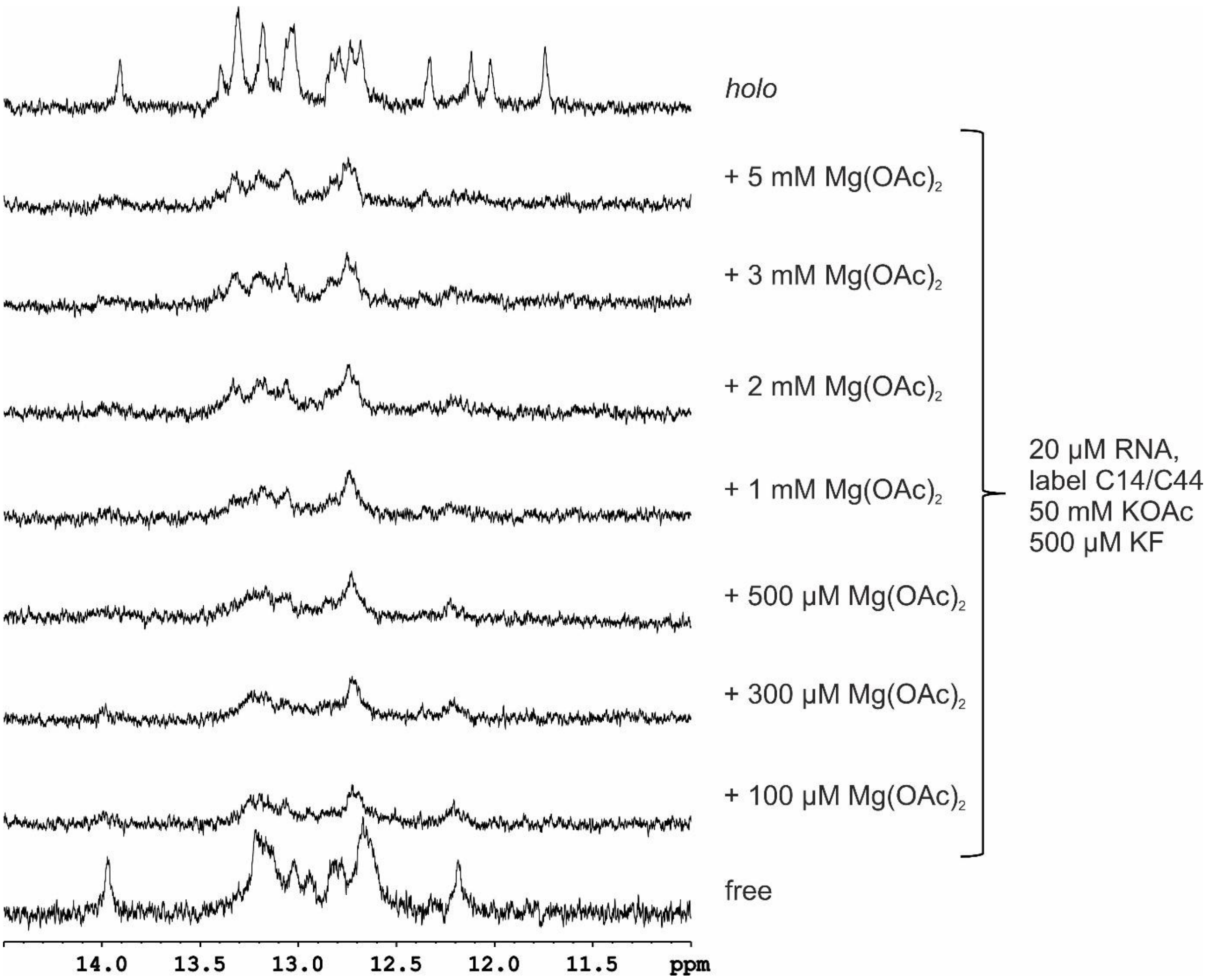

Figure S10: Imino region of the ^{1}H NMR spectra for the free (bottom spectrum) and holo (top spectrum) form of the unlabelled fluoride riboswitch and the titration series of the fluoride riboswitch labelled in positions C14/C44 with 50 mM KOAc, 5 mM $Mg(OAc)_2$, increasing amount of Mg^{2+} as indicated in the figure, 10% D_2O. The buffer composition of the free form was 50 mM KOAc, 10% D_2O and for the holo form 50 mM KOAc, 5 mM $Mg(OAc)_2$, 500 µM KF, 10% D_2O. Spectra were measured at 700 MHz and 295 K.

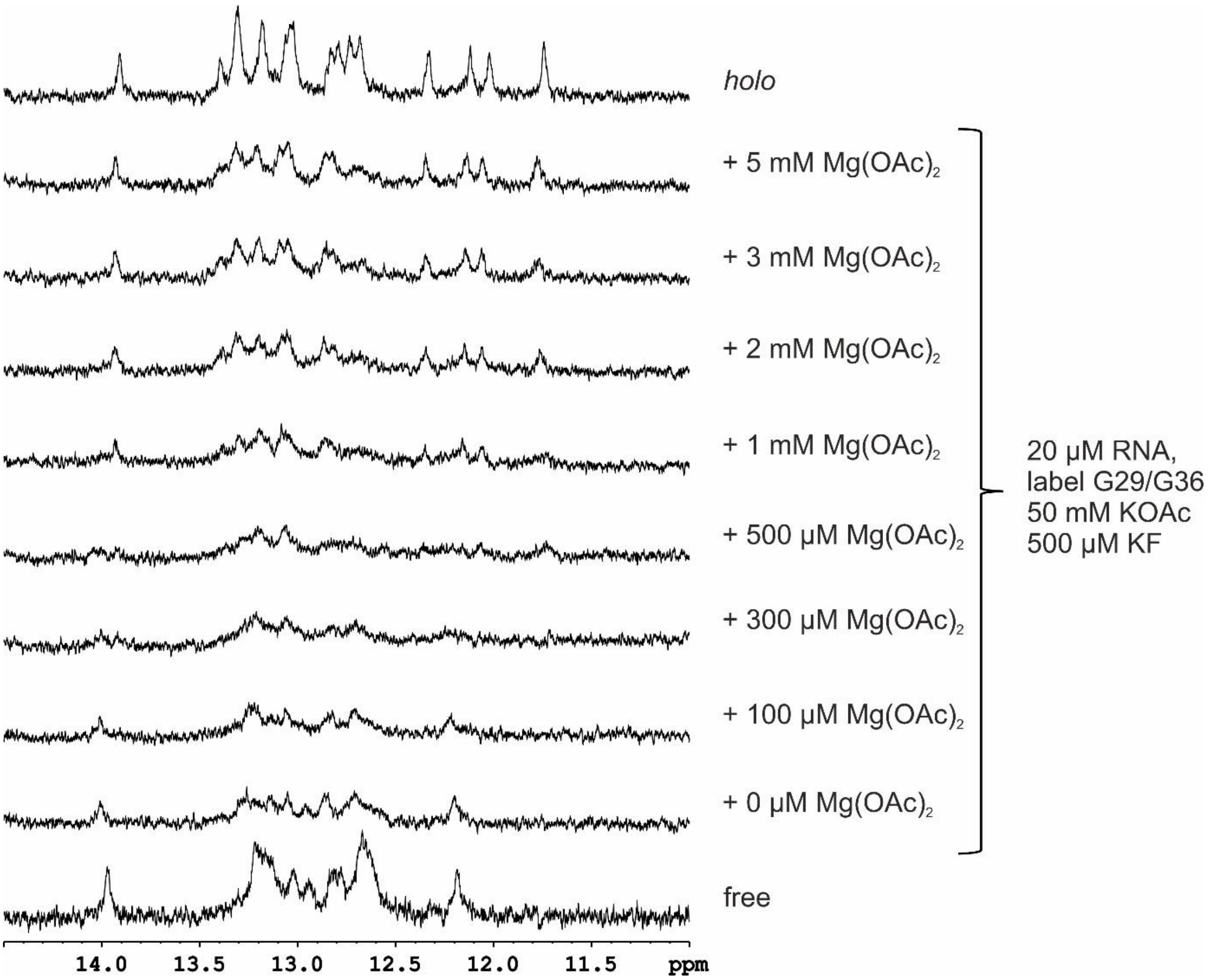

Figure S11: Imino region of the ^1H NMR spectra for the free (bottom spectrum) and the holo (top spectrum) form of the unlabelled fluoride riboswitch and the titration series of the fluoride riboswitch labelled in positions G29/G36 with 50 mM KOAc, 5 mM $Mg(OAc)_2$, increasing amount of Mg^{2+} as indicated in the figure, 10% D_2O. The buffer composition of the free form was 50 mM KOAc, 10% D_2O and for the holo form 50 mM KOAc, 5 mM $Mg(OAc)_2$, 500 µM KF, 10% D_2O. Spectra were measured at 700 MHz and 295 K.

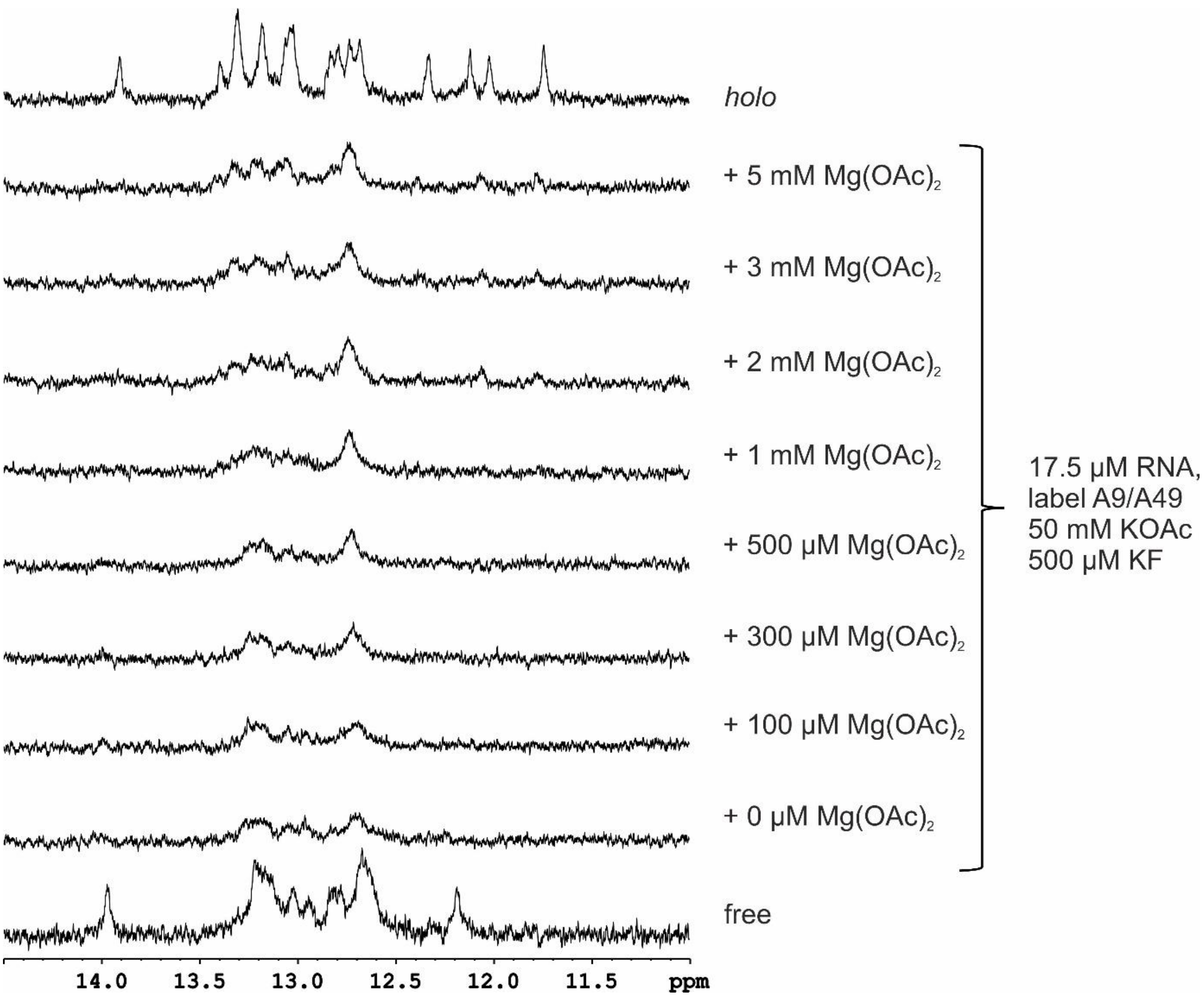

Figure S12: Imino region of the ^1H NMR spectra for the free (bottom spectrum) and the holo (top spectrum) form of the unlabelled fluoride riboswitch and the titration series of the fluoride riboswitch labelled in positions A9/A49 with 50 mM KOAc, 5 mM Mg(OAc)$_2$, increasing amount of Mg^{2+} as indicated in the figure, 10% D_2O. The buffer composition of the free form was 50 mM KOAc, 10% D_2O and for the holo form 50 mM KOAc, 5 mM Mg(OAc)$_2$, 500 µM KF, 10% D_2O. Spectra were measured at 700 MHz and 295 K.

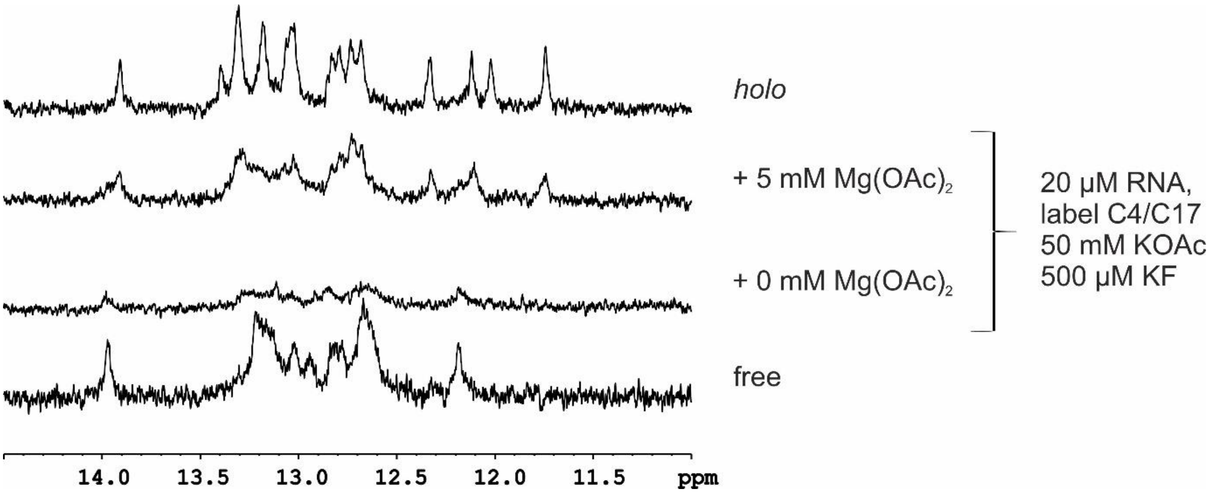

Figure S13: Imino region of the ^1H NMR spectra for the free (bottom spectrum) and the holo (top spectrum) form of the unlabelled fluoride riboswitch the fluoride riboswitch labelled in positions C4/C17 with 50 mM KOAc, 5 mM Mg(OAc)$_2$, no or 5 mM Mg^{2+} as indicated in the figure, 10% D_2O. The buffer composition of the free form was 50 mM KOAc, 10% D_2O and for the holo form 50 mM KOAc, 5 mM Mg(OAc)$_2$, 500 µM KF, 10% D_2O. Spectra were measured at 700 MHz and 295 K.

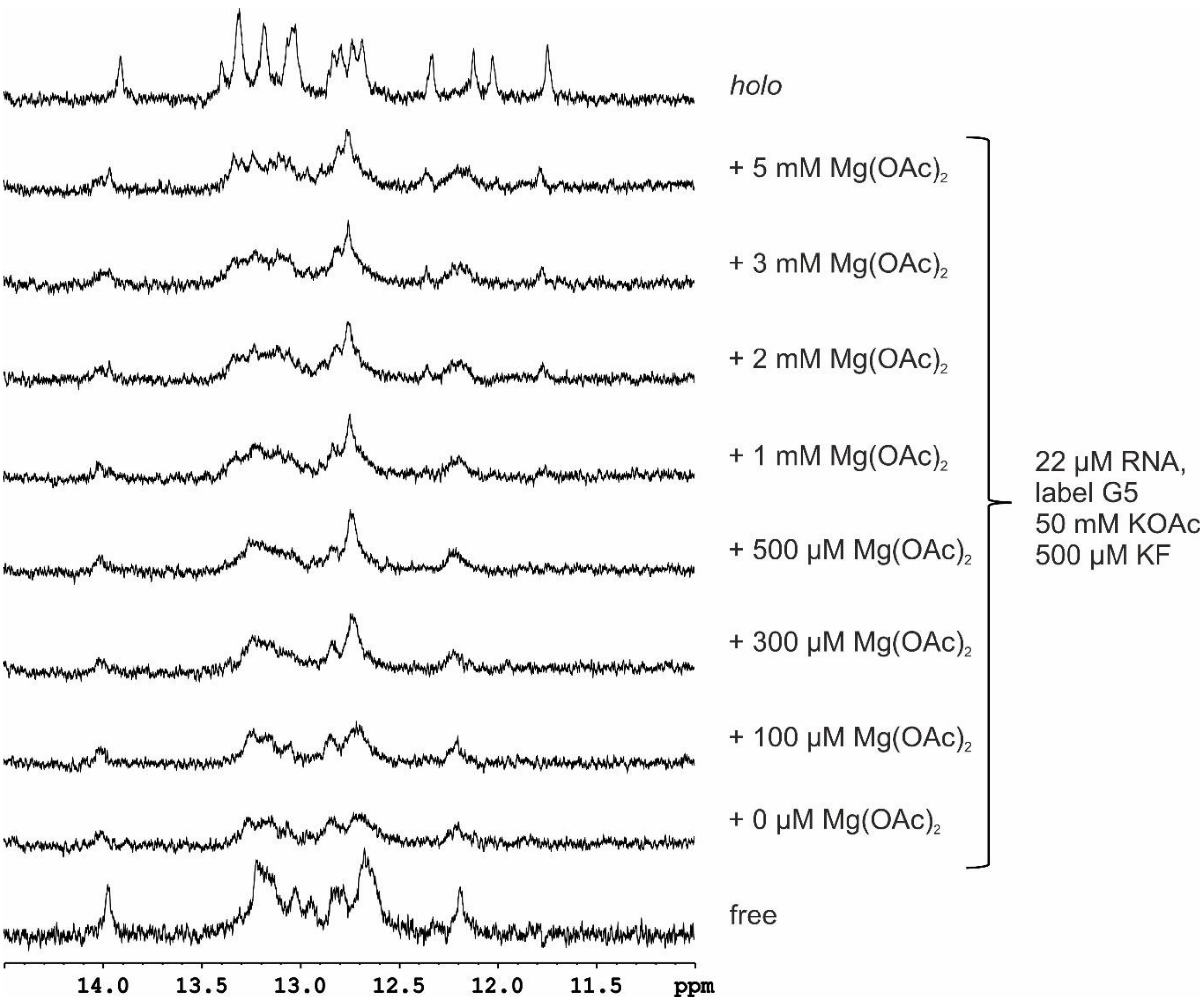

Figure S14: Imino region of the ^1H NMR spectra for the free (bottom spectrum) and the holo (top spectrum) form of the unlabelled fluoride riboswitch and the titration series of the fluoride riboswitch labelled in positions G5 with 50 mM KOAc, 5 mM $Mg(OAc)_2$, increasing amount of Mg^{2+} as indicated in the figure, 10% D_2O. The buffer composition of the free form was 50 mM KOAc, 10% D_2O and for the holo form state 50 mM KOAc, 5 mM $Mg(OAc)_2$, 500 µM KF, 10% D_2O. Spectra were measured at 700 MHz and 295 K.

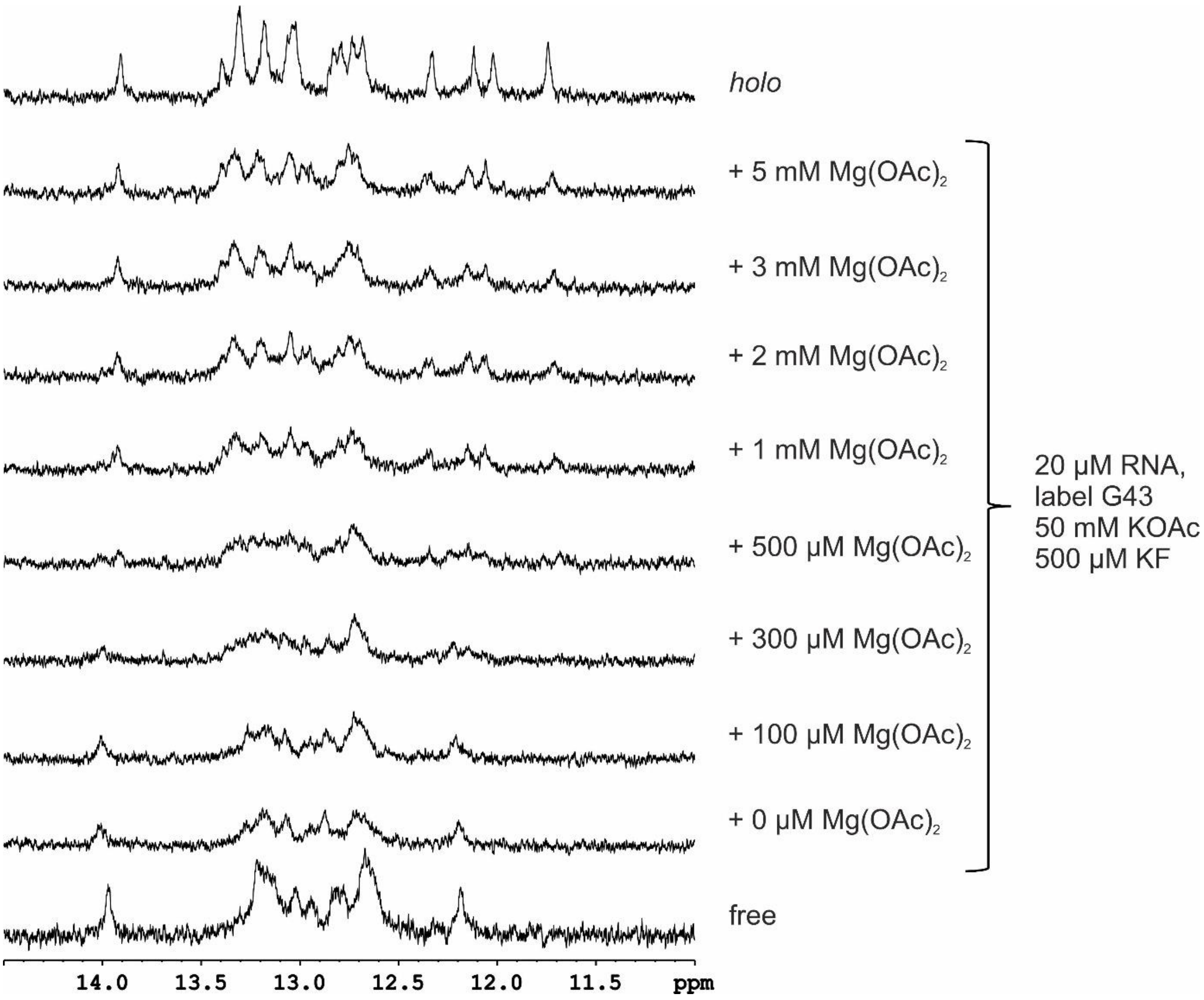

Figure S15: Imino region of the ^{1}H NMR spectra for the free (bottom spectrum) and the holo (top spectrum) form of the unlabelled fluoride riboswitch and the titration series of the fluoride riboswitch labelled in positions G43 with 50 mM KOAc, 5 mM $Mg(OAc)_2$, increasing amount of Mg^{2+} as indicated in the figure, 10% D_2O. The buffer composition of the free form was 50 mM KOAc, 10% D_2O and for the holo form 50 mM KOAc, 5 mM $Mg(OAc)_2$, 500 µM KF, 10% D_2O. Spectra were measured at 700 MHz and 295 K.

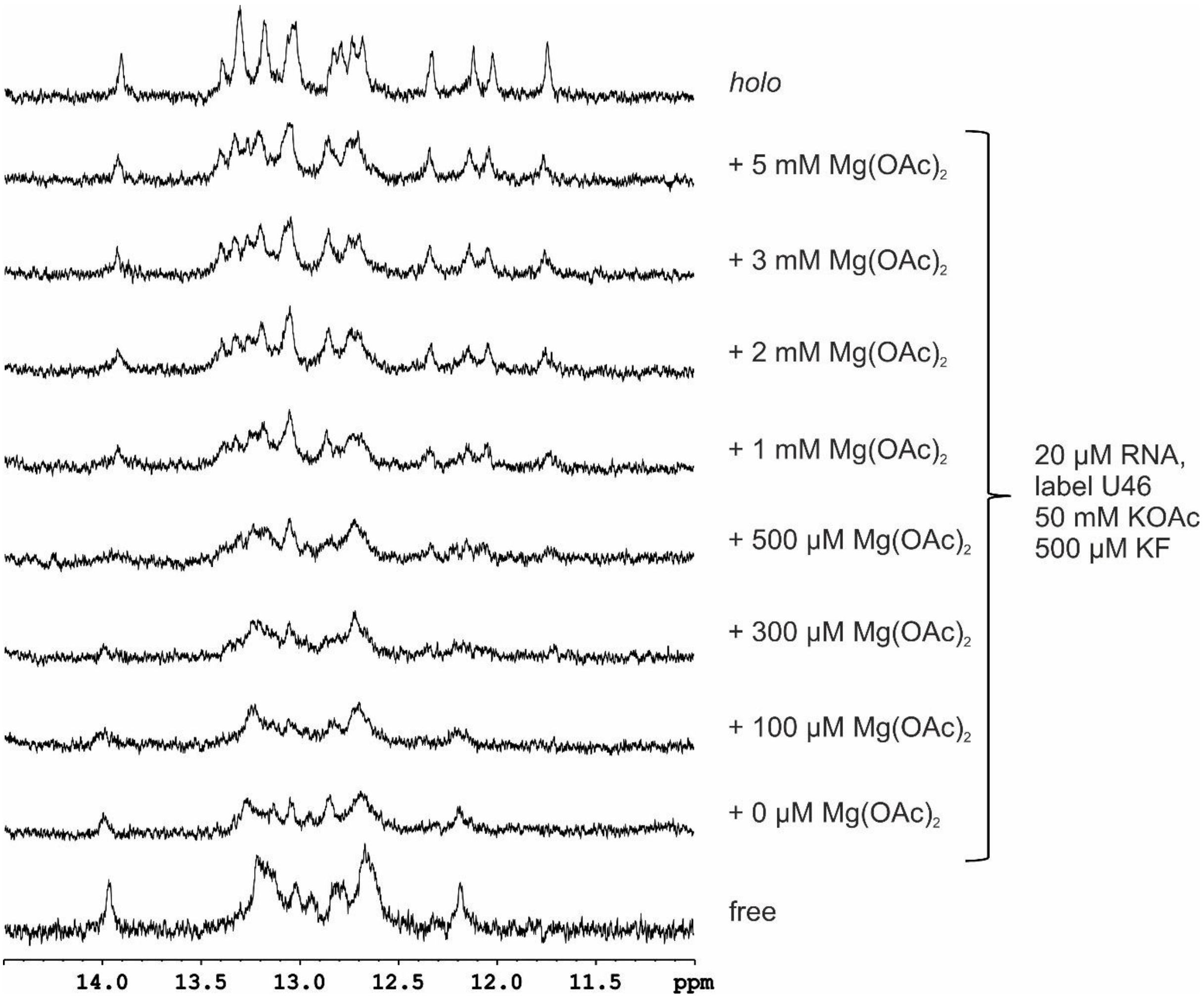

Figure S16: Imino region of the ^1H NMR spectra for the free (bottom spectrum) and the holo (top spectrum) form of the unlabelled fluoride riboswitch and the titration series of the fluoride riboswitch labelled in positions U46 with 50 mM KOAc, 5 mM $Mg(OAc)_2$, increasing amount of Mg^{2+} as indicated in the figure, 10% D_2O. The buffer composition of the free form was 50 mM KOAc, 10% D_2O and for the holo form 50 mM KOAc, 5 mM $Mg(OAc)_2$, 500 µM KF, 10% D_2O. Spectra were measured at 700 MHz and 295 K.

5. Control Experiments and analysis for PELDOR

5.1. PELDOR analysis on doubly labelled constructs

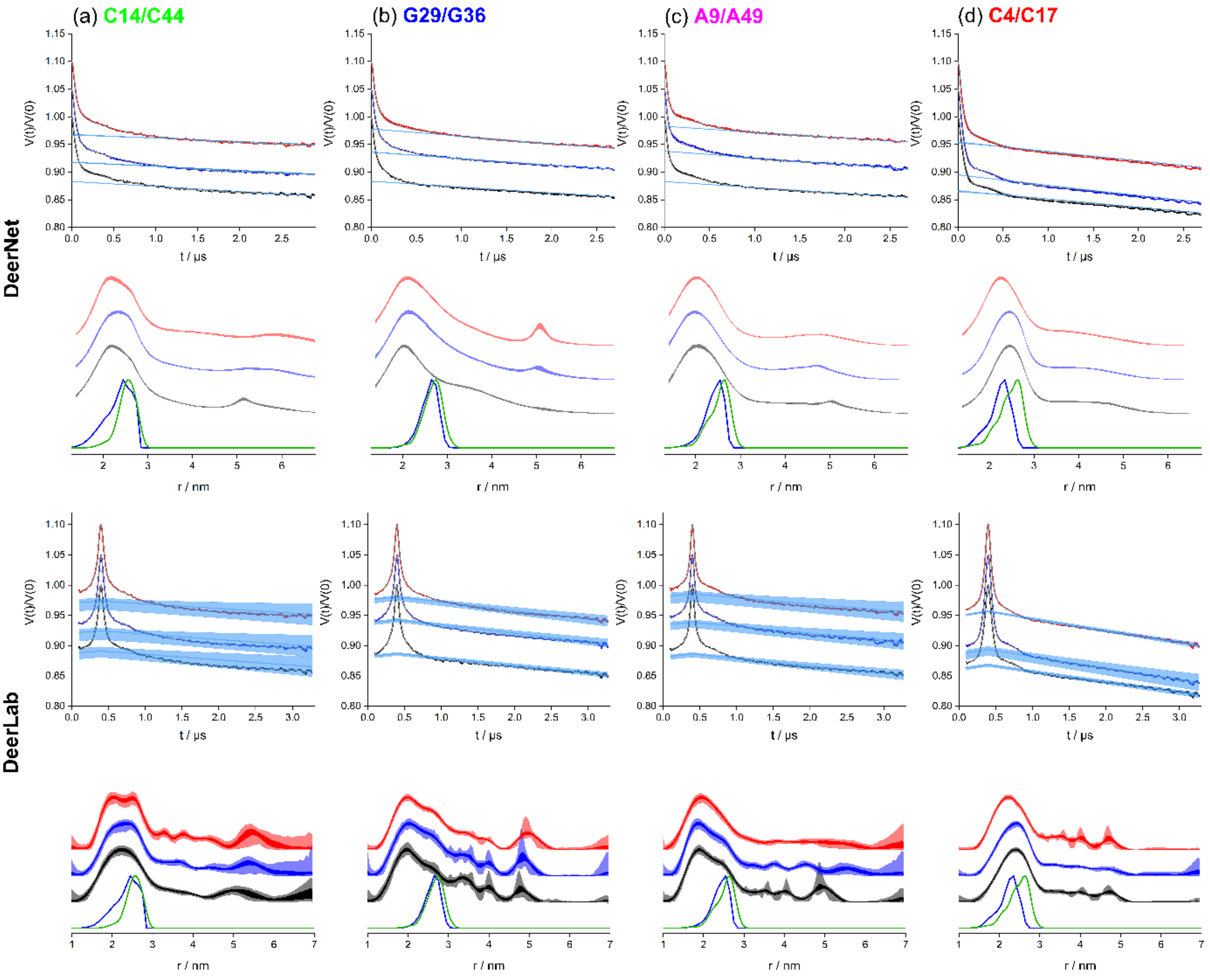

Figure S17: PELDOR time traces and distance distributions of the fluoride riboswitch labelled at positions C14/C44 (a), G29/G36 (b), A9/A49 (c), C4/C17 (d) in the free (black time trace/area), apo (blue time trace/area), and holo (red time trace/area) forms. Time traces were measured at Q-band MW frequency and 50 K. Analysis was performed with DeerNet (first and second row) and DeerLab (third and fourth row). The background of the time traces is represented in light blue. The error of the DeerNet distributions is given by the width of the distance distribution. For the DeerNet distance distributions the 50% (dark grey/blue/red) and the 95% (light grey/blue/red) confidence intervals are given. The distance distributions from in silico labelling with the MtsslSuite (blue line) and MMM (green line) are given for comparison. A vertical offset was introduced for both, the time traces and the distance distributions.

To ensure that the high similarity of the distance distributions for the different forms of the fluoride riboswitch obtained from PELDOR measurements (see Figure 2 in main text) is not due to user bias in the processing with DeerAnalysis2022, the processing was also performed with DeerNet and DeerLab. Figure S17 shows the time traces with the background and the corresponding distance distributions from the DeerNet and the DeerLab output.

5.2. PELDOR measurements using a constructs with one spin label

To exclude that multiple riboswitch molecules are stacking, causing multi-spin effects and an additional distance in the PELDOR measurement, a PELDOR measurement with a single spin label per riboswitch molecule was performed at different RNA concentrations (10 µM (a) and 235 µM (b)). If there was stacking, a significant modulation depth should be visible. Compared to the modulation depths seen in the doubly-labelled samples, the modulation depth shown here can be neglected and no reliable distance could be observed, showing that stacking is unlikely (Figure S18).

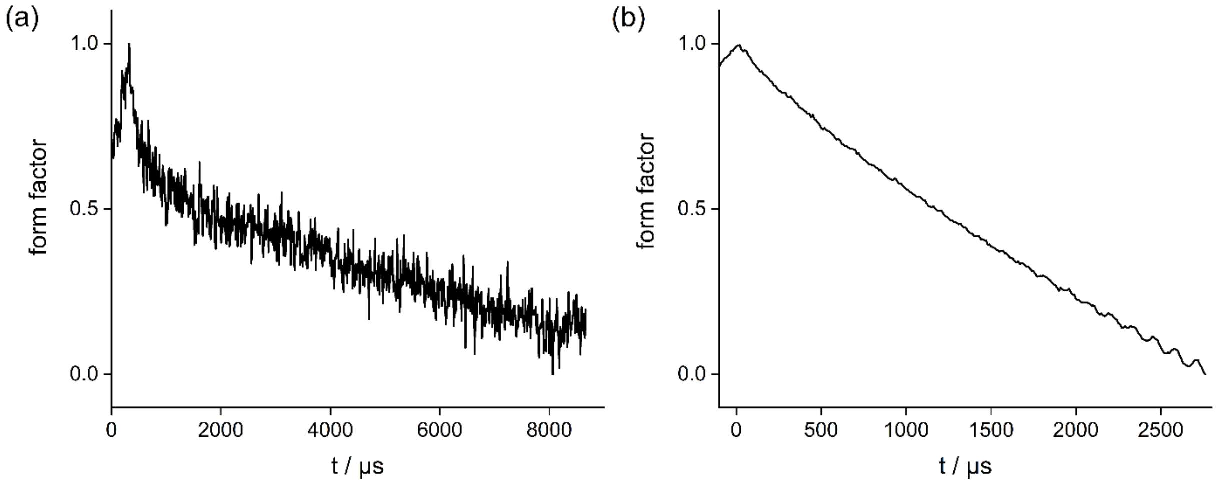

Figure S18: PELDOR time traces of the fluoride riboswitch labelled at position U46 (10 µM (a), 235 µM (b)) in 50 mM KOAc, 5 mM $Mg(OAc)_2$, 500 µM KF, 20% glycerol-d_8 recorded at Q-band.

6. Control Experiments and analysis for ^{19}F ENDOR

6.1. Echo detected EPR measurements

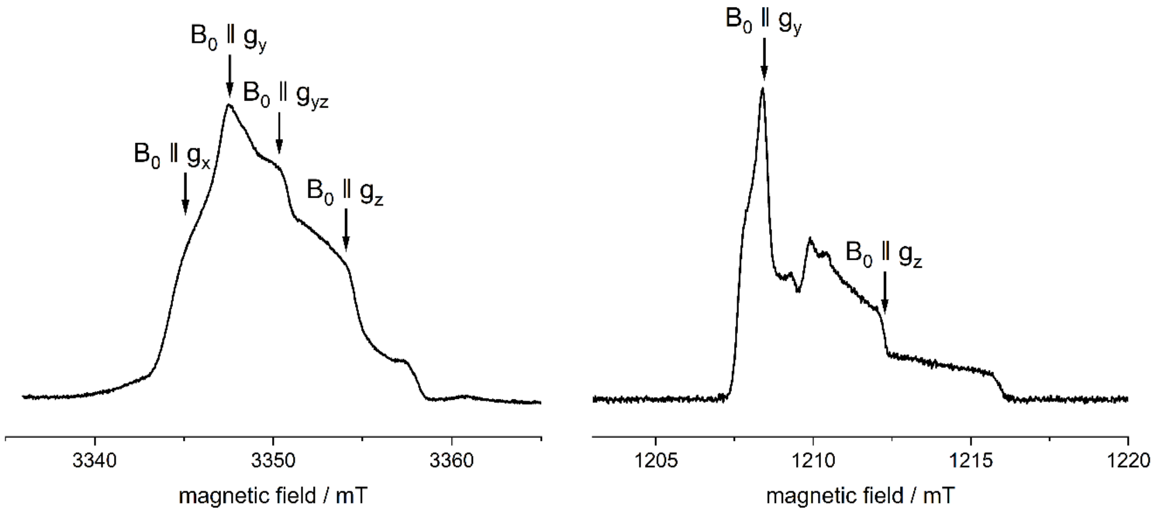

Figure S19: Echo detected EPR spectra of the riboswitch in the holo form labelled in position G5 and G43 measured at W-band and 50 K (left) and U46 measured at Q-band and 50 K (right). Field positions for measurements of Mims ^{19}F ENDOR spectra are indicated by arrows. The RNA concentration was adjusted to 100 µM, 150 µM, and 235 µM RNA for the RNA labelled in position G5, G43, and U46, respectively. For W-band measurements a protonated spin label and for Q-band measurements a deuterated spin label was used.

6.2. ^{19}F ENDOR measurements of U46 at W-band

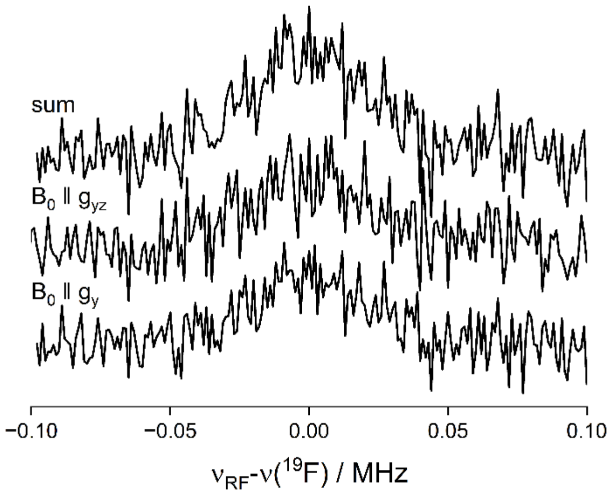

Figure S20: 94 GHz (W-band) Mims ^{19}F ENDOR spectra of the fluoride riboswitch in the holo form labelled in position U46 at different field positions as indicated in the figure. The temperature was set to 50 K. The RNA concentration was adjusted to 150 µM. A protonated spin label was used.

6.3. ^{19}F ENDOR measurements at Q-band with protonated spin label

When measuring ^{19}F ENDOR spectra of a sample with a protonated spin label at Q-band MW frequency a significant overlap of the ^{19}F ENDOR signal with the signal originating from the protons of the spin label can be observed (Figure S21). This additional signal leads to a distortion of the ^{19}F ENDOR signal, making the evaluation difficult. Therefore, it is necessary to replace the protonated spin label with a deuterated equivalent.

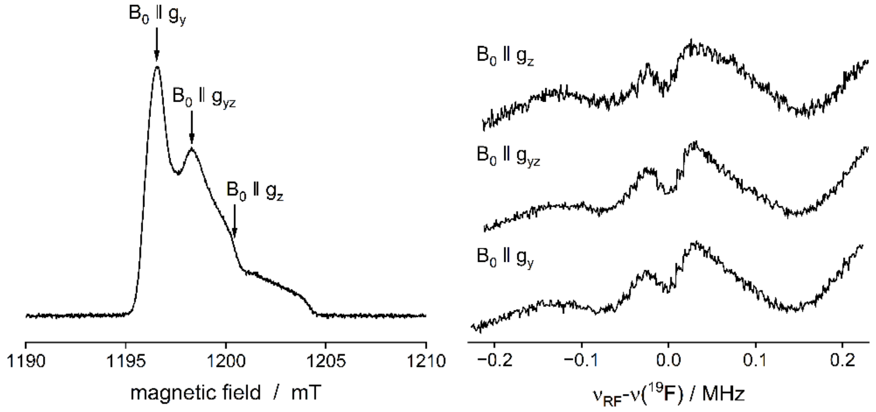

Figure S21: Echo detected EPR spectrum of the riboswitch in the holo form labelled with protonated spin label in position G5 (left) and ^{19}F ENDOR spectra at different field positions as indicated in the field swept EPR spectrum (right). Measurements were performed at Q-band MW frequency and 50 K. The RNA concentration was adjusted to 100 µM. Significant contribution from the overlap of the ^{19}F ENDOR signal with the proton signal can be observed.

6.4. T_M measurements

The phase memory time T_M has been determined for all ENDOR samples and the delay T has been chosen according to the length of the RF pulse used in the ENDOR experiment. For the sample with the deuterated spin label (U46 in red) a slower relaxation can be observed compared to the samples with protonated spin label (G5 in black and G43 in blue) at the same T (60 µs). For the sample with the deuterated spin label ^{19}F ENDOR measurements have also been performed with 200 µs RF pulses. The corresponding T_M trace is shown in purple.

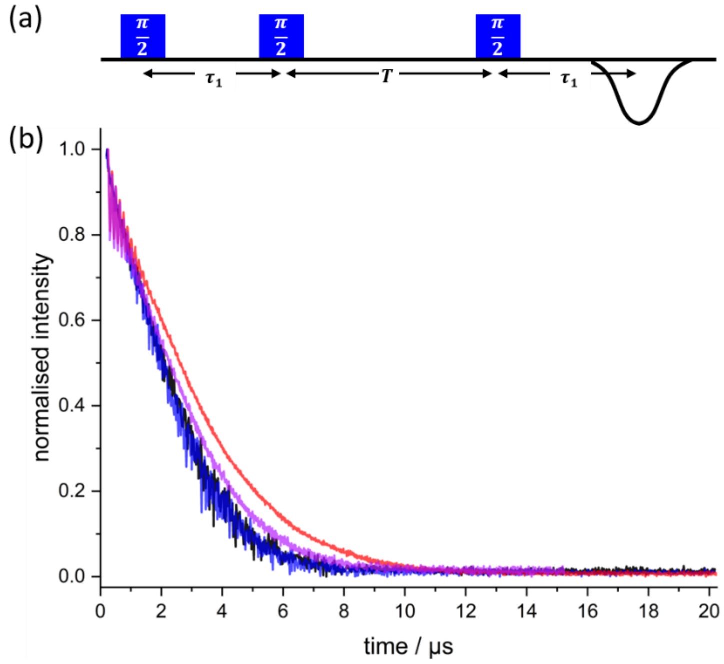

Figure S22: (a) Pulse sequence of the 3-pulse echo decay measurement used for the determination of the phase memory time T_M; (b) T_M measurements of the fluoride binding riboswitch labelled with protonated spin label in position G5 with a delay T = 60 µs (black), labelled with protonated spin label in position G43 with a delay T = 60 µs (blue), labelled with deuterated spin label in position U46 with a delay T = 60 µs (red), and labelled with deuterated spin label in position U46 with a delay T = 210 µs (purple). Measurements were performed at W-band MW frequency (G5 and G43) and Q-band MW frequency (U46). The temperature was set to 50 K. The RNA concentration was adjusted to 100 µM (G5), 150 µM (G43), and 235 µM (U46).

6.5. Background measurement

To show that the free F^- in the sample does not cause a background signal in the ^{19}F ENDOR spectra a measurement of fluoride riboswitch labelled in position G43 (protonated label) and U46 (deuterated label) in presence of F^- but without Mg^{2+} has been performed at W-band and Q-band, respectively. In these samples, F^- cannot bind to the riboswitch since the Mg^{2+}-cluster cannot be formed but the nitroxide label attached to the RNA will still be able to interact with the free F^- in solution. Figure S23 shows the ^{19}F ENDOR spectrum of the RNA sample without Mg^{2+} for G43 at W-band (a) and U46 at Q-band (b). This shows that there is no background signal arising from a potential interaction of the spin label with the free F^-.

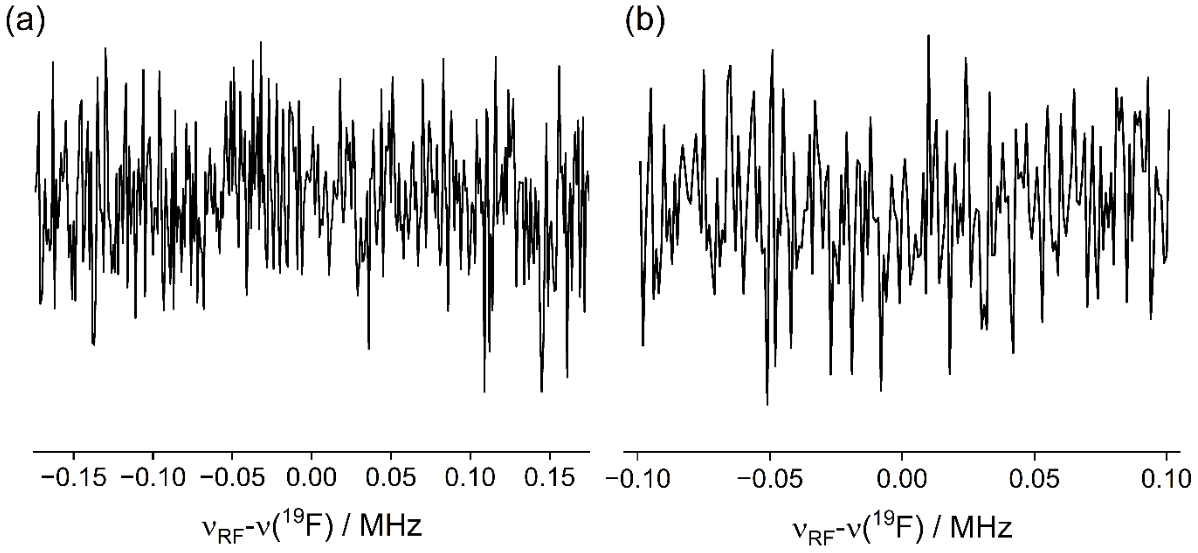

Figure S23: (a) Mims ^{19}F ENDOR spectrum for G43 in absence of Mg^{2+}. The sample was composed of 150 µM RNA labelled in position G43, 50 mM KOAc, 500 µM KF, and 20% glycerol-d_8. The measurement was performed at W-band MW frequency and 50 K; (b) Mims ^{19}F ENDOR spectrum for U46 in absence of Mg^{2+}. The sample was composed of 175 µM RNA labelled in position U46, 50 mM KOAc, 500 µM KF, and 20% glycerol-d_8. The measurement was performed at Q-band MW frequency and 50 K. For both measurements the field position was set to the maximum of the field swept EPR spectrum. Measurements were performed over night.

6.6. ^{19}F ENDOR measurements at different field positions

Figure S24 shows the ^{19}F ENDOR spectra of the fluoride riboswitch labelled at position G5 (a), G43 (b), and U46 (c) measured at the different field positions shown in the field swept EPR spectrum (Figure S19) and the sum spectrum. T_{read} is highlighted with red dotted lines.

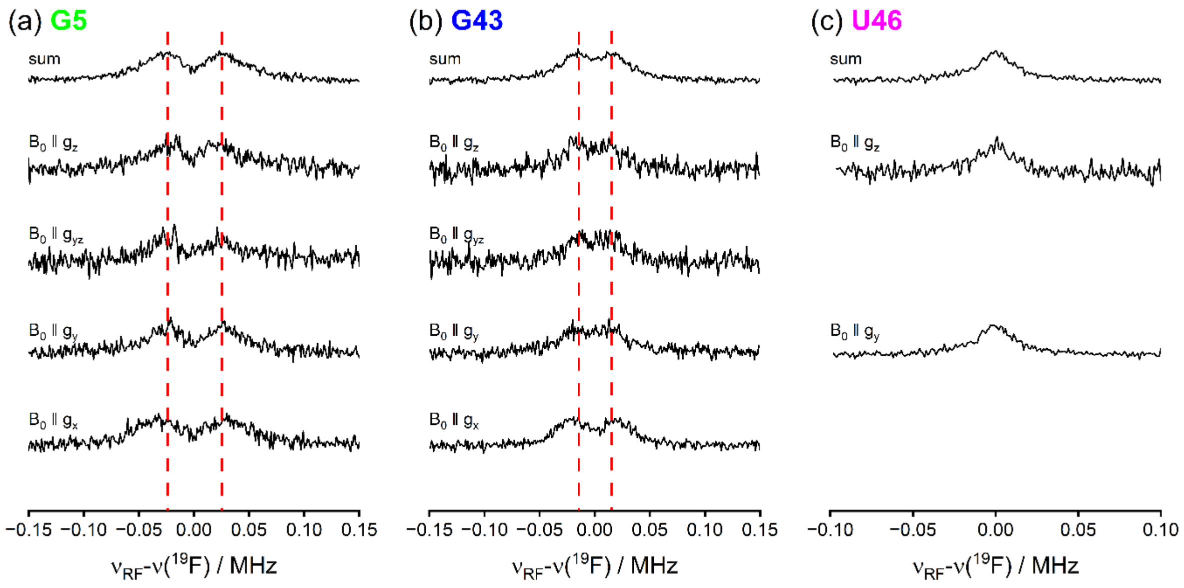

Figure S24: Mims ^{19}F ENDOR spectra (black) of the fluoride riboswitch in the holo form labelled in positions G5, G43, and U46 at different field positions (field swept EPR spectra compare Figure S19) as indicated in the figure. The coupling constant determined from the spectrum for the respective construct is highlighted by dashed lines. Measurements were performed at W-band MW frequency (G5 and G43) and Q-band MW frequency (U46). The temperature was set to 50 K. The RNA concentration was adjusted to 100 µM (G5), 150 µM (G43), and 235 µM (U46). For W-band measurements a protonated spin label and for Q-band measurements a deuterated spin label was used.

6.7. Estimation of coupling constant and width of Gaussian distribution of distances

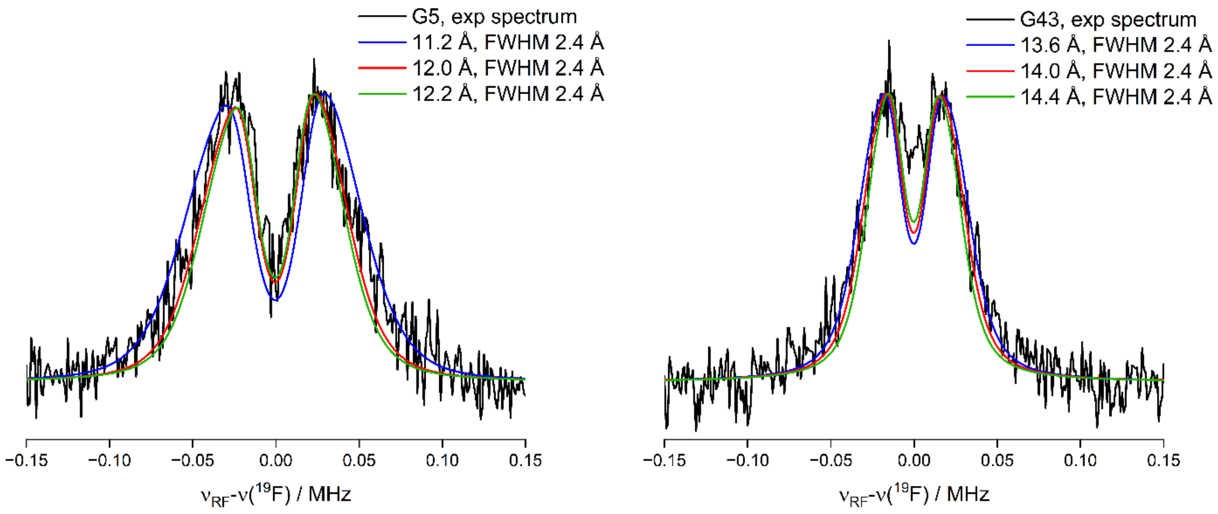

Figure S25: Simulation of the ^{19}F ENDOR spectrum of G5 (left, black) and G43 (right, black) using Gaussian distance distributions with different mean distances as indicated in the figure (coloured lines) to estimate the error of R_{sim}.

Table S4: RMSD values for the simulated sum spectra of G5 and G43 using Gaussian distance distributions of different mean distance and FWHM of 2.4 Å.

Construct	Mean distance R_{sim}	RMSD
G5	11.2 Å	1.05E-01
	12.0Å	9.04E-02
	12.2 Å	9.73E-02
G43	13.6 Å	9.85E-02
	14.0 Å	9.20E-02
	14.4 Å	9.53E-02

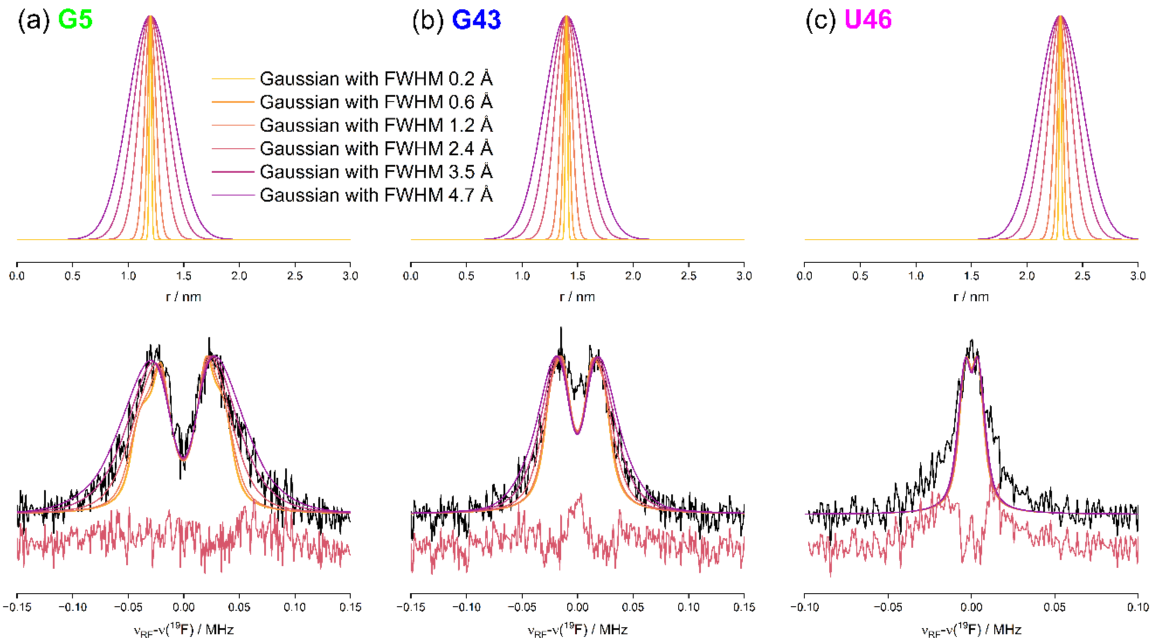

Figure S26: Simulation of the ^{19}F ENDOR sum spectrum of the three constructs G5, G43, and U46 (black) using Gaussian distributions of distances with different FWHM as indicated in the figure (coloured lines). Residuals for the simulations with the Gaussian distributions of distances for each sum spectrum are given below the respective spectrum.

Table S 5: RMSD values for the simulated sum spectra using Gaussian distance distributions of different FWHM. For U46 no RMSD was determines as this value would not be representative.

Construct	FWHM	RMSD
G5	0.2 Å	1.28E-01
	0.6 Å	1.25E-01
	1.2 Å	1.15E-01
	2.4 Å	9.04E-02
	3.5 Å	8.17E-02
	4.7 Å	1.03E-01
G43	0.2 Å	1.06E-01
	0.6 Å	1.05E-01
	1.2 Å	1.01E-01
	2.4 Å	9.20E-02
	3.5 Å	9.28E-02
	4.7 Å	1.09E-01

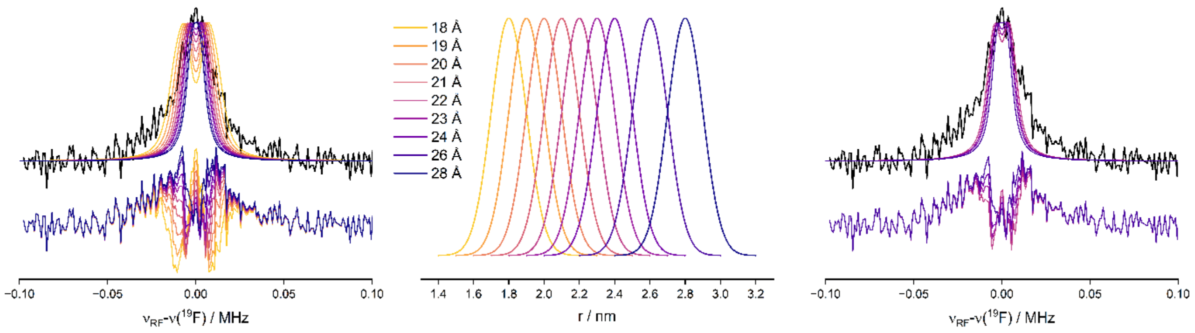

Figure S27: Simulation of the ^{19}F ENDOR spectrum of U46 using Gaussian distance distributions with different mean distances and the respective residuals (left), the corresponding Gaussian distance distributions (centre), and the simulations indicating the lower distance limit with no more resolved splitting from 23 Å (22-26 Å, right) and the respective residuals using a linewidth of 7 kHz.

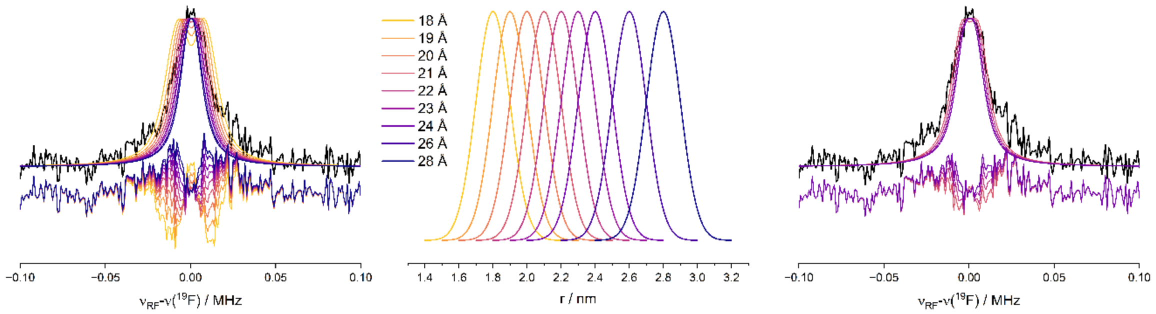

Figure S 28: Simulation of the ^{19}F ENDOR spectrum of U46 using Gaussian distance distributions with different mean distances and the respective residuals (left), the corresponding Gaussian distance distributions (centre), and the simulations indicating the lower distance limit with no more resolved splitting from 21 Å (21-24 Å, right) and the respective residuals using a linewidth of 12 kHz.

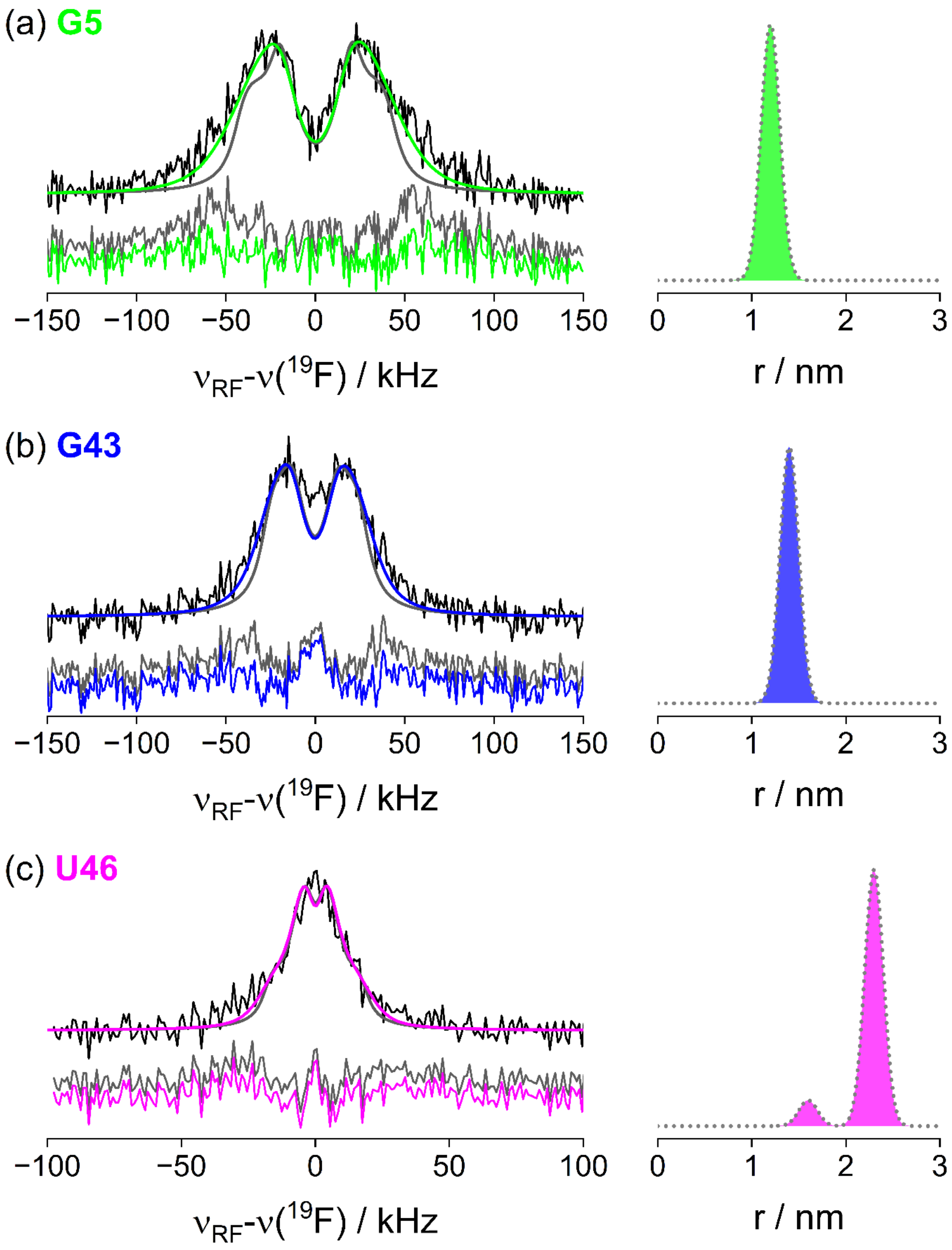

Figure S29: Mims ^{19}F ENDOR sum spectra of the holo aptamer and simulated spectra (left) using Gaussian distributions (right, coloured) or based on T_{read} (grey, compare main text) and the respective residuals.

6.8. Analysis with rotamers from MtsslSuite and MMM

For the fluoride riboswitch labelled in position U46 the ^{19}F ENDOR spectra could not be simulated using all rotamers (the whole distance distribution) obtained from *in silico* spin labelling in either a 1:1 ratio (MtsslSuite) or with the given population factors (MMM) since it would result in a resolved splitting in the simulated spectrum for both (Figure S30). This is due to the large contribution of the diastereomer resulting in the short distances (red/orange). These lead to a coupling constant not that prominent in the measured spectra of the actual RNA sample. Taking into consideration only the distances arising from the diastereomer leading to the long contributions of the distance distribution, the ^{19}F ENDOR spectra can be better described by the simulation (blue/green). Since MMM does account for both diastereomeric labelling positions by default, in the evaluation rotamers with a distance shorter or longer than 16.5 Å were considered for the respective cases. This distance corresponds to the local minimum between the two main distance contributions of the distance distribution obtained from MMM.

It can be seen here, that for the simulations with the diastereomer resulting in the longer distance the spectrum can still not be fully reproduced. A low population of a shorter distance seems to be present. Since MtsslSuite does not determine a population factor of the rotamers a small contribution of the diastereomer resulting in the short distance was considered to account for this feature in the measured spectrum (Fig. 4, main text).

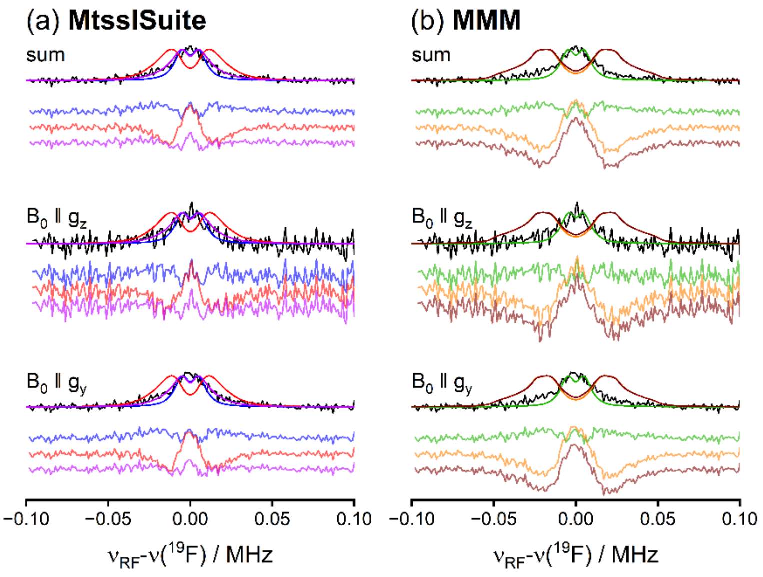

Figure S30: Mims ^{19}F ENDOR spectra (black) of the fluoride riboswitch in the holo form labelled in position U46 at different field positions (echo detected EPR spectrum compare Figure S19) as indicated in the figure. The RNA concentration was adjusted to 235 µM RNA. Measurements were performed at Q-band MW frequency. A deuterated spin label was used. Simulations of the ^{19}F ENDOR spectra were performed with SimSpec[19] using the distances and orientations of the rotamers obtained from the in silico labelling with MtsslSuite (a) or MMM (b). Comparison of simulations using only the short contributions of the distance distribution (red/orange), only the long contributions of the distance distribution (blue/green) or the whole distance distribution (purple/brown) and the corresponding rotamers.

7. Analysis of the riboswitch structure

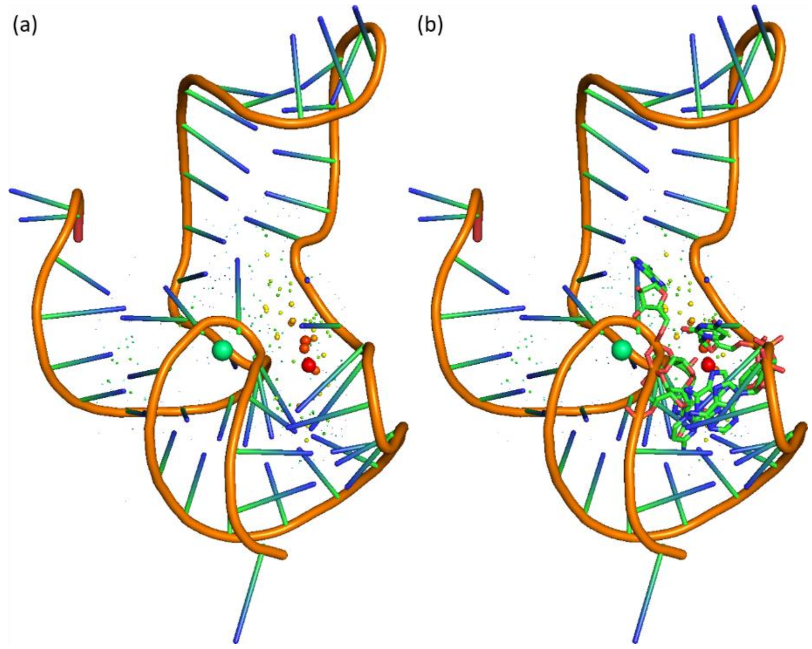

Figure S31: Trilateration of the F^- position based on the crystal structure of the fluoride riboswitch from T. petrophila (PDB: 4ENC)[20] *performed with MtsslSuite. The position of the F^- in the crystal structure (green sphere) is given for comparison. The probability cloud with the most probable (red), less likely (orange/yellow) and unlikely (green) F^- positions are shown without (a) and with graphical representation of the surrounding bases (b). The likeliness of the F^- position is increasing with increasing sphere size. Parameters are given in Section 1.4.*

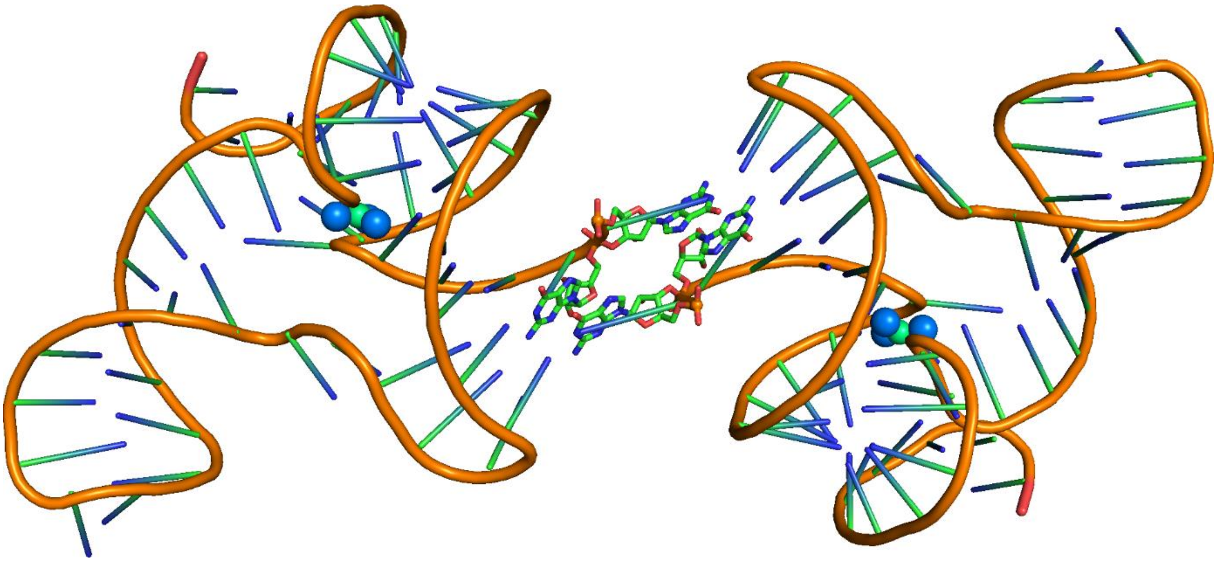

Figure S32: Graphical representation of the crystal contacts between residues G1 and G2 of two fluoride riboswitch units (PDB: 4ENC).[20]

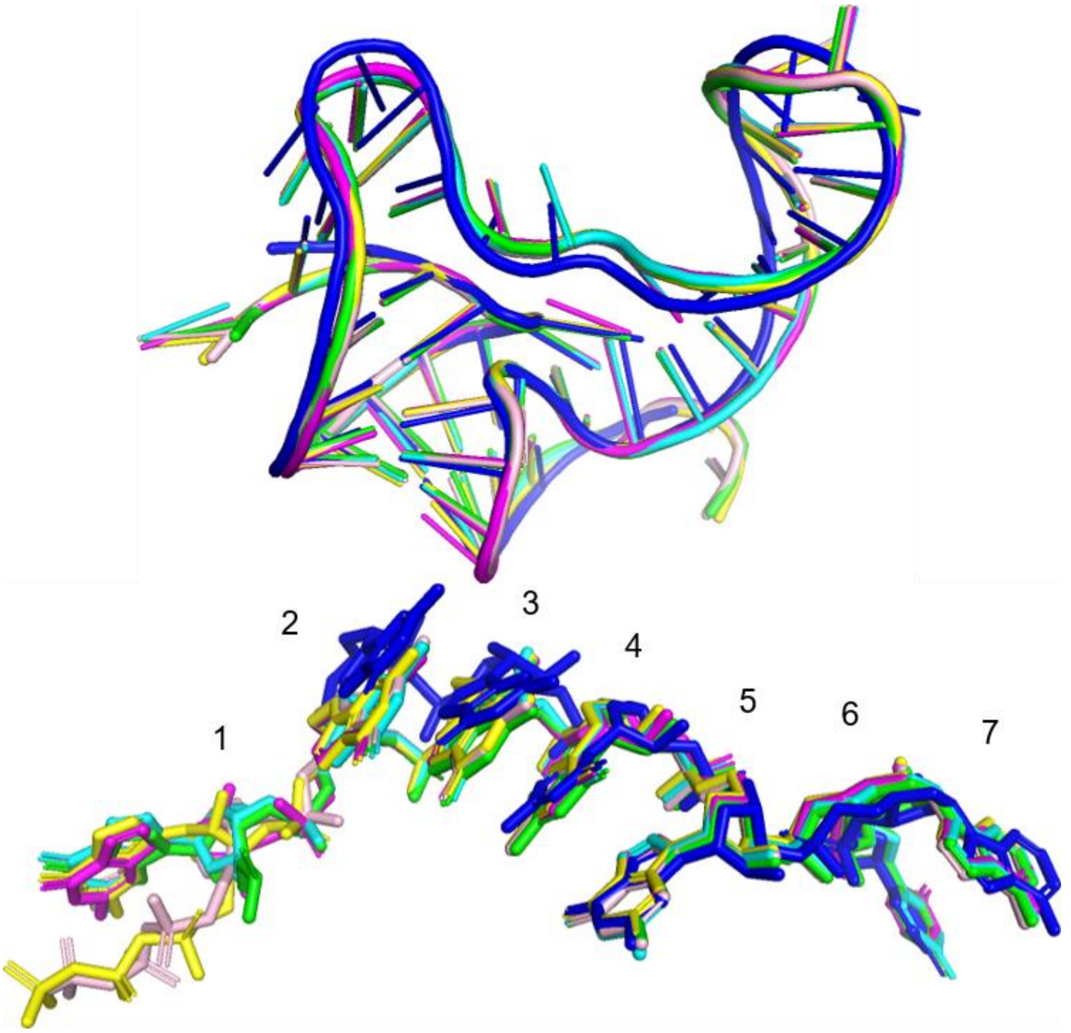

Figure S33: Overlay of the whole structure and residues 1-7 of the T. petrophila fluoride riboswitch structures 4ENC (green), 3VRS (cyan), 4EN5 (magenta), 4ENA (yellow), 4ENB (light pink), and residues 1-6 of the B. cereus fluoride riboswitch structure 5KH8 (dark blue).[20,21]

References

[1] G. Hagelueken, R. Ward, J. H. Naismith, O. Schiemann, *Appl. Magn. Reson.* **2012**, *42*, 377–391.

[2] G. Hagelueken, D. Abdullin, R. Ward, O. Schiemann, *Mol. Phys.* **2013**, *111*, 2757–2766.

[3] G. Hagelueken, D. Abdullin, O. Schiemann, in *Methods Enzymol.* (Eds.: P.Z. Qin, K.B.T.-M. in E. Warncke), Academic Press, **2015**, pp. 595–622.

[4] G. Jeschke, *Protein Sci.* **2018**, *27*, 76–85.

[5] Y. Polyhach, E. Bordignon, G. Jeschke, *Phys. Chem. Chem. Phys.* **2011**, *13*, 2356–2366.

[6] S. D. Pirrwitz J., *Verfahren Zur Herstellung von 2,2,6,6-Tetramethyl-4-Oxopiperidin*, **1984**, DD222017A (WP C 07 D/260 901 6).

[7] L. A. Shundrin, I. A. Kirilyuk, I. A. Grigor'ev, *Mendeleev Commun.* **2014**, *24*, 298–300.

[8] M. M. Haugland, A. H. El-Sagheer, R. J. Porter, J. Peña, T. Brown, E. A. Anderson, J. E. Lovett, *J. Am. Chem. Soc.* **2016**, *138*, 9069–9072.

[9] G. Úr, T. Kálai, K. Hideg, *Tetrahedron Lett.* **2016**, *57*, 778–780.

[10] P. L. Beaulieu, P. C. Anderson, D. R. Cameron, G. Croteau, V. Gorys, C. Grand-Maître, D. Lamarre, F. Liard, W. Paris, L. Plamondon, F. Soucy, D. Thibeault, D. Wernic, C. Yoakim, S. Pav, L. Tong, *J. Med. Chem.* **2000**, *43*, 1094–1108.

[11] P. Z. Qin, I. S. Haworth, Q. Cai, A. K. Kusnetzow, G. P. G. Grant, E. A. Price, G. Z. Sowa, A. Popova, B. Herreros, H. He, *Nat. Protoc.* **2007**, *2*, 2354–2365.

[12] T. L. Hwang, A. J. Shaka, *J. Magn. Reson. Ser. A* **1995**, *112*, 275–279.

[13] M. Pannier, S. Veit, A. Godt, G. Jeschke, H. W. Spiess, *J. Magn. Reson.* **2000**, *142*, 331–340.

[14] C. E. Tait, S. Stoll, *Phys. Chem. Chem. Phys.* **2016**, *18*, 18470–18485.

[15] G. Jeschke, V. Chechik, P. Ionita, A. Godt, H. Zimmermann, J. Banham, C. R. Timmel, D. Hilger, H. Jung, *Appl. Magn. Reson.* **2006**, *30*, 473–498.

[16] K. Ackermann, C. Pliotas, S. Valera, J. H. Naismith, B. E. Bode, *Biophys. J.* **2017**, *113*, 1968–1978.

[17] S. G. Worswick, J. A. Spencer, G. Jeschke, I. Kuprov, *Sci. Adv.* **2018**, *4*, eaat5218.

[18] L. Fábregas Ibáñez, G. Jeschke, S. Stoll, *Magn. Reson.* **2020**, *1*, 209–224.

[19] H. Wiechers, A. Kehl, M. Hiller, B. Eltzner, S. F. Huckemann, A. Meyer, I. Tkach, M. Bennati, Y. Pokern, *J. Magn. Reson.* **2023**, *353*, 107491.

[20] A. Ren, K. R. Rajashankar, D. J. Patel, *Nature* **2012**, *486*, 85–89.

[21] B. Zhao, S. L. Guffy, B. Williams, Q. Zhang, *Nat. Chem. Biol.* **2017**, *13*, 968–974.
